# Per‐ and polyfluoroalkyl substances in land‐applied biosolids: Accumulation in soils, crop uptake, and potential dietary risk

**DOI:** 10.1002/jeq2.70220

**Published:** 2026-07-15

**Authors:** Summer Streets, Emerson F. C. Souza, Matthew McNearney, Sona Jedinak, Alonso Doria Manzur, Jennifer L. Guelfo, Carl Rosen

**Affiliations:** ^1^ Minnesota Pollution Control Agency Duluth Minnesota USA; ^2^ Department of Soil, Water, and Climate University of Minnesota Saint Paul Minnesota USA; ^3^ Department of Civil, Environmental, and Construction Engineering Texas Tech University Lubbock Texas USA

## Abstract

Applying biosolids as a soil amendment for crop production is a common practice, raising concerns about the introduction of per‐ and polyfluoroalkyl substances (PFAS) into soils and crops at application sites. We investigated the impact of biosolids application on PFAS concentrations in agricultural soils and crops at three different farms that received biosolids from three different wastewater treatment plants in Minnesota. At each farm, three fields were identified: one with no history of biosolids application, one with past biosolids application, and one with biosolids application in the study year (2023). Biosolids; soils; and corn (*Zea mays*), rye (*Secale cereale*), and soybeans (*Glycine max*) were analyzed for PFAS. Biosolids‐amended soils showed a greater number of PFAS, as well as higher total PFAS concentrations than fields that had never received biosolids or had not received biosolids in recent years. Several PFAS, including perfluorooctane sulfonate (PFOS), were found to accumulate in corn, especially in the stalks and leaves (stover). PFAS were also found in rye, but not in soybeans. Bioaccumulation factors for crops were calculated for perfluorobutane sulfonate (5.0–5.2, corn stover), perfluorobutanoic acid (0.57, rye, and 7.0–7.7, corn stover), perfluoropentanoic acid (0.34, corn stover), and PFOS (0.03, corn stover). Crop and soil results were used to perform a human health risk assessment using a simple exposure model to estimate PFOS concentration in beef and milk. Hazard quotients were >1 for both adults and children based on 90th percentile consumption rates, indicating a potential for human health risk in the modeled scenario.

## Core Ideas

- Soils amended with biosolids had more per‐ and polyfluoroalkyl substances (PFAS) than soils with no history of biosolids use.
- Most PFAS were retained in surficial soil (0–30 cm).
- PFAS were detected in corn stover and rye but not corn ears or soybeans.
- Corn stover grown on biosolids‐treated land may pose a risk to beef and milk consumers.

## Plain Language Summary

Per‐ and polyfluoroalkyl substances (PFAS) are a large group of chemicals with many industrial and consumer uses. PFAS may harm humans and the environment, raising concerns about impacts on soils, crops, and livestock as after using biosolids as a soil treatment, which is a common practice across the United States. Biosolids have been shown to contain PFAS. Here, we measured PFAS in biosolids, soils, and crops at three working farms in Minnesota. Our objective was to understand how applying biosolids containing PFAS would affect the amount of PFAS in soil and crops. We used our data to estimate potential risk to adults and children consuming beef and milk of cattle fed a diet with the same amount of PFAS found in our study. We found that soils amended with biosolids had more PFAS than soils at the same sites with no history of biosolids use. PFAS was found in corn stover, and rye grains, but not corn kernels or soybean grains. Corn stover grown on biosolids‐treated fields may pose a risk to those who eat beef and milk.

**Abbreviations**
PFBA: perfluorobutanoic acid
PFBS: perfluorobutane sulfonate
PFCA: perfluoroalkyl carboxylate
PFNA: perfluorononanoic acid
PFOA: perfluorooctanoic acid
PFOS: perfluorooctane sulfonate
PFPeA: perfluoropentanoic acid
PFSA: perfluoroalkyl sulfonate

## 1 INTRODUCTION

Per‐ and polyfluoroalkyl substances (PFAS) are a large and diverse group of persistent, bioaccumulative, and toxic chemicals widely used in numerous industrial processes and consumer goods (Glüge et al., 2020). PFAS are very resistant to degradation due to their carbon‐fluorine bond, which is highly stable, leading to their lasting presence in the environment (Openiyi et al., 2024; Oza et al., 2024). PFAS have a number of demonstrated and suspected adverse effects on human health including immune system suppression, impacts on endocrine organs (breasts, ovaries, testes, and thyroid), reproductive and developmental harm, kidney disorders, and a variety of cancers (Habib et al., 2024). PFAS have been detected in multiple environmental media worldwide, including biosolids (Behnami et al., 2024; Panieri et al., 2022), agricultural soils (Brusseau et al., 2020), and crops (Biswas et al., 2025; Lesmeister et al., 2021).

Numerous studies have demonstrated that biosolids applied to agricultural fields as soil amendments are a source of PFAS in agricultural soils (Alvarez‐Ruiz et al., 2024; Garza‐Rubalcava et al., 2026; Klamerus et al., 2025; Moodie et al., 2021; Oviedo‐Vargas et al., 2025; L. G. Peter, Lee, et al., 2025; L. Peter, Modiri‐Gharehveran, et al., 2025; Schaefer et al., 2022; Smith et al., 2024). However, there are very few field studies available measuring PFAS in biosolids, soils, and plants on working farms over the course of a growing season, and even fewer studies evaluating PFAS soil concentration and plant uptake in multiple fields within a single farm with a range of biosolids application (no history, past application, current application). In addition, studies describing PFAS soil and plant concentration at farms receiving biosolids with relatively low PFAS concentrations (i.e., not industrially impacted) are lacking.

One of the primary concerns related to the use of PFAS‐laden biosolids as a soil amendment is the potential for uptake of PFAS into crops. While plant uptake of PFAS is still a growing area of research, several studies have demonstrated that PFAS uptake does occur in crops (Adu et al., 2023; Costello & Lee, 2020; Lesmeister et al., 2021) leading to potential livestock and human exposure (Death et al., 2021; Jha et al., 2021; Maddela et al., 2022). In a controlled study, six dairy cows (Holsteins) were given PFAS‐contaminated feed for 28 days (Kowalczyk et al., 2013). Perfluorooctane sulfonate (PFOS) was found to be highly bioaccumulative in tissues (blood, liver, muscle), and 14% of accumulated PFOS was ultimately excreted in milk. In an investigation of the bioaccumulation of PFAS in dairy cows that received contaminated feed and drinking water, Vestergren et al. (2013) found that consumption of contaminated silage was the dominant exposure pathway, and biotransfer factors (BTFs) derived in that study demonstrated a high potential for transfer of PFAS to meat and milk. Results from a physiologically based pharmacokinetic model that described uptake of PFAS from feed and subsequent elimination demonstrated that, while the elimination rate of PFOS from dairy cows is low, almost all eliminated PFOS is excreted through milk (van Asselt et al., 2013). Overall, shorter‐chain PFAS are not accumulated in dairy cows to the same degree as longer‐chain PFAS (Lupton et al., 2014). For example, perfluorobutane sulfonate (PFBS) was shown to be readily excreted and did not accumulate in dairy cows (Kowalczyk et al., 2013).

While much attention is given to human exposure to PFAS via drinking water, diet is a major route of human exposure for some PFAS, particularly outside of contaminated communities where drinking water does not dominate exposure. For example, diet was estimated to contribute between 66% and 100% of total PFOS exposure, with other exposure pathways (water, dust, product use, and inhalation) accounting for the remaining exposure contributions (De Silva et al., 2021). Likewise, a study by Poothong et al. (2020) characterized exposure to PFAS via multiple routes, demonstrating that ingestion of food and drink were the most significant exposure routes for humans. The European Food Safety Authority panel on contaminants in the food chain (EFSA CONTAM Panel, 2020) determined that the contribution of meat and meat products to mean PFOS exposure was exceeded in importance only by that of fish/seafood and eggs.

The objective of this study was to conduct a comprehensive field assessment evaluating the impacts of land‐applied biosolids on PFAS contamination in agricultural soils and crop accumulation of PFAS under conditions typical of commercial agricultural settings in Minnesota. To accomplish this objective, we tested soil samples collected during four sampling events in a single growing season, and crop tissue collected at harvest, from multiple farm fields representing a range of biosolid treatment conditions, soil types, and crops. Based on previous studies, we expected that fields receiving biosolids would have higher concentrations and a greater number of PFAS, and thus, greater potential for plant uptake. Site data were coupled with a simple exposure model to determine potential risks to human consumers of beef and milk produced by hypothetical livestock fed a diet consisting of silage (represented by corn [*Zea mays*] stover). Although field data were collected in Minnesota, the results presented here are relevant across the United States, particularly where soil types and biosolids application rates are comparable.

## 2 MATERIALS AND METHODS

### 2.1 Site selection

Three wastewater treatment plants (WWTPs) and three farms volunteered to participate in this study in 2022–2023. For each paired WWTP–farm location (labeled as locations B, H, and I), three fields were selected (nine total fields), each with a unique history of biosolids application. At each of the three farms, field 1 had no record of biosolids application, field 2 had biosolids applied 2 or more years prior to the study year, and field 3 had biosolids applied during the study year in either fall of 2022 or spring of 2023. It is important to note that biosolids application occurred *at least* 2 years prior to the study at all field 2 locations, however, the last application could have been many years ago. Information detailing prior biosolids application at all field 2 locations, including timing of biosolids application and application rate, was not available. Due to the sensitive nature of PFAS detections in wastewater and farmland, the locations of the fields sampled are not provided, but elevations, relative areas, and the distance between fields at each location are shown in Figure S1. All crops were grown under rainfed conditions, and 409, 520, and 389 mm of precipitation were recorded during the growing season (April to October) at locations B, H, and I, respectively. On average, the seasonal 30‐year (1993–2022) historical precipitation at these sites were 587, 583, and 670 mm (Minnesota State Climatology Office, 2024), indicating that the 2023 growing season was drier than average.

**Core Ideas**

- Soils amended with biosolids had more per‐ and polyfluoroalkyl substances (PFAS) than soils with no history of biosolids use.
- Most PFAS were retained in surficial soil (0–30 cm).
- PFAS were detected in corn stover and rye but not corn ears or soybeans.
- Corn stover grown on biosolids‐treated land may pose a risk to beef and milk consumers.

### 2.2 Sample collection

Biosolids from each WWTP were collected for PFAS analysis and nutrient content at the time of application at each facility/location pair. Application rates of biosolids at all sites were based on meeting the nitrogen (N) requirements for corn production (Kaiser et al., 2023). Details related to WWTP treatment type and application rates for each site is provided in the Supporting Information.

Soil samples were collected at three depths (0–30, 30–60, and 60–90 cm) in each field using a clean 2.5 cm diameter stainless steel tube. At each depth, six cores were collected at various points and thoroughly homogenized in a pre‐cleaned bucket with a pre‐cleaned spade to make a composite soil sample for each field at each depth. An aliquot of each composite soil sample was submitted for analysis. This process was performed three times to create a triplicate set of composite samples at each depth and in each field. The 0‐ to 30‐cm increment was sampled first, and the tube rinsed thoroughly in the field with PFAS‐free water provided by the analytical lab before sampling at the next depth (30–60 cm) and rinsed again before sampling at the 60‐ to 90‐cm increment. Boreholes were filled with soil from the site immediately following sample collection.

Four sampling events were conducted during the growing season: preplanting (April 2023), early growing season (June 2023), mid‐growing season (August 2023), and postharvest (October 2023). Soil samples were collected at three depths at each sampling event except for August when only at two depths (0–30 and 30–60 cm) were collected. The GPS location of each soil sample was recorded to allow for soil sampling and crop harvesting in approximately the same areas. Samples were placed in PFAS‐free polypropylene bags and stored at 4°C until the time of analysis. Equipment preparation and sampling precautions are described in the Supporting Information.

Plant samples were immediately placed in clean, uncoated brown paper bags and stored in a cooler for transport to the lab. Plants were not allowed to touch the ground after harvesting. Corn ears (grain and cobs) and stover (stalks and leaves), rye (*Secale cereale*) grain heads, and soybean (*Glycine max*) pods were dried at 60°C in brown paper bags in a drying oven prior to shipment to the analytical lab. None of the harvested crops were washed at any point in the handling process. Soybeans were separated from pods after drying and prior to shipping to the analytical lab. Rye grain heads and corn ears were shipped to the lab intact. Corn was the only crop grown on fields that received biosolids in the study year. Details of soil and crop sampling, including the total number of samples, type of crop grown at each field, number of crop samples, and types of analyses performed on both soils and crops is presented in Table S1.

### 2.3 PFAS analysis

Soil and crop samples, along with quality assurance/quality control (QA/QC) samples (field blanks, trip blanks, equipment rinse blanks, and sample duplicates) were analyzed by Eurofins Lancaster Laboratories Environment Testing, LLC (Eurofins) for 40 PFAS using EPA method 1633 (US EPA, 2024). Biosolids samples were analyzed for 46 PFAS by Texas Tech University (TTU) using methods previously published (Sepulvado et al., 2011; Shojaei et al., 2022). Details of TTU methods can be found in Tables S2 and S3. A table comparing Eurofins and TTU PFAS analyte lists and limits of quantitation of the laboratories used in this study can be found in Table S4. Additional information on TTU extraction methods is provided in the Supporting Information. Prior to the start of this study, ultrapure laboratory deionized water and four conventional fertilizer samples were tested for PFAS at SGS Axys Analytical Laboratories in Sydney, British Columbia, Canada for PFAS analysis using EPA method 1633. Those data are provided in Table S5 for context but are not discussed in detail in this article.

### 2.4 Other analyses

Soil samples were analyzed by the University of Minnesota Research Analytical Lab for soil texture, total organic carbon, organic matter, soil pH, nutrients, and USEPA 503 metals. Biosolids samples were tested for nutrients, total N, ammonia and other nutrients, pH, total solids, and USEPA 503 metals. Plants were tested for essential nutrients and trace metals. Data are provided in Table S5, and methods are described in the Supporting Information. However, these results are not discussed in the article as no correlations between any of these parameters and PFAS concentrations were identified.

### 2.5 QA/QC and data acceptance

All soil samples were collected in triplicate. In addition, duplicate samples were collected and analyzed for PFAS at a rate of approximately 10% of total samples collected. One aqueous field blank and one aqueous trip blank were prepared at each sampling event and analyzed for PFAS. PFAS‐free water was provided by Eurofins for preparation of field blanks, which were prepared by opening the provided water in the field and decanting into a clean sample container while following all sampling precautions. Trip blanks were prepared by Eurofins and transported to the sample sites in a cooler with the other sample containers.

Extractable internal standard recovery <20% resulted in rejection of the corresponding congener in the sample. For laboratory control samples (LCS and LLCS [low‐level laboratory control samples]) the acceptable recovery range was 25%–150%. Sample results flagged with a “B” by the laboratory, indicating the compound was present in the method blank, were rejected if the reported sample concentration was <3x the reported blank concentration. Estimated data qualified with a “J flag” (concentration between the method detection limit (MDL) and reporting limit) or “I” flag (estimated possible maximum concentration) were included in final calculations. In cases where a specific PFAS compound was detected in only one of the three triplicate samples, that single detection was counted only in the assessment of the total number of PFAS detections but was otherwise excluded from further analysis and interpretation.

### 2.6 Data interpretation, visualization, and statistical analysis

Summary statistics and figures were generated in Microsoft Excel. For analytes that were detected in two of three samples in a set of triplicates, half the MDL was used in place of non‐detects to calculate mean concentration. This approach to non‐detects is supported by EPA (US EPA, 1991).

Bioaccumulation factors (BAFs) were calculated as the ratio of the mean concentration of a given PFAS in crop samples to the mean concentration of that same PFAS in soil (Equation 1).

(1) $$\mathrm{BAF}=\frac{C_{\mathrm{crop}}}{C_{\mathrm{soil}}}$$

where *C*_crop_ is the mean concentration of a PFAS in crop tissue and *C*_soil_ is the mean concentration of a PFAS in soil

The BAF was derived for each PFAS detected in both vegetation and corresponding soil samples.

### 2.7 Human health risk assessment

A simple exposure model was used to estimate the total PFOS concentration in beef muscle and milk based on PFOS transfer to feed (Figures S2 and S3). The assessment is described in detail in the Supporting Information, and all parameters used can be found in Table S6. In brief, the model exposure of cattle to PFAS in feed (corn silage represented by an average wet weight corn stover [stalk and leaves] concentration and haylage), soil, and water. While corn silage is typically composed of stover and ears, corn ear samples for location I, field 3 were all non‐detect for PFOS and were therefore excluded from the assessment. The risk to humans was then estimated based on children (ages 1 to <6) and adults (age 20+) consuming beef and milk using mean and 90th percentile consumption rates for both groups. The beef or milk screening level represents the maximum amount of PFOS in beef or milk that people can safely consume. Only noncancer risk estimates (hazard quotients [HQs]) are provided as they are more stringent than estimates based on cancer risk.

## 3 RESULTS AND DISCUSSION

### 3.1 Biosolids

Up to 23 PFAS were detected in biosolids samples B, H, and I, collectively. Concentrations ranged from 0.355 ng g^−1^ dry weight (dw) perfluorononanoic acid (PFNA; biosolid B) to 280 ng g^−1^ dw PFBS (biosolid H; Table 1). Biosolids from facility I had the greatest number of PFAS (20), followed by H (19), and B (11). Biosolid H had the highest ƩPFAS (total mass‐based PFAS concentration) at (371 ng g^−1^ dw), which was largely driven by PFBS (280 ng g^−1^ dw). ƩPFAS was 185 ng g^−1^ dw in biosolid I, and 51.1 ng g^−1^ dw in biosolid B. Perfluoropentane sulfonate was >50% of the ƩPFAS in biosolid B (27.2 ng g^−1^ dw). Biosolid I had the highest PFOS concentration (49.5 ng g^−1^ dw), compared to 7.14 ng g^−1^ dw in biosolid H and 3.59 ng g^−1^ dw in biosolid B. The range of PFAS concentrations in biosolids from these facilities suggests differences in source contribution.

**TABLE 1:** Per‐ and polyfluoroalkyl substances (PFAS) concentrations in biosolids and corresponding detection frequencies in field 3 soil samples across all locations.

Group	Number of carbon atoms	CAS number	Analyte	Biosolid B April 2023 (ng g^−1^ dw)	Biosolid H November 2023 (ng g^−1^ dw)	Biosolid I April 2023 (ng g^−1^ dw)	Detection frequency in soil (%)
PFCA	4	375‐22‐4	PFBA	1.96	23.4	3.15	67
	5	2706‐90‐3	PFPeA	0.748	2.42	7.01	26
	6	307‐24‐4	PFHxA	0.596	8.82	8.64	39
	7	375‐85‐9	PFHpA	<1.0	0.880	0.927	33
	8	335‐67‐1	PFOA	0.847	4.10	4.79	68
	9	375‐95‐1	PFNA	0.355	0.817	3.79	30
	10	335‐76‐2	PFDA	1.00	2.16	20.9	28
	11	2058‐94‐8	PFUnA	<1.0	0.876	2.34	7
	12	307‐55‐1	PFDoA	<2.0	1.49	5.85	14
	13	72629‐94‐8	PFTrDA	<0.5	<0.5	1.48	4
	14	376‐06‐7	PFTeDA	<1.0	<1.0	1.82	6
PFSA	4	375‐73‐5	PFBS	<0.2	280	8.83	41
	5	2706‐91‐4	PFPeS	27.2	9.34	8.17	0
	6	335‐46‐4	PFHxS	6.02	8.37	<2.0	9
	7	375‐92‐8	PFHpS	<0.2	<0.2	3.85	3
	8	1763‐23‐1	PFOS	3.59	7.14	49.5	58
	10	335‐77‐3	PFDS	<2.0	<2.0	3.08	12
FASA	8	754‐91‐6	PFOSA	<1.0	1.79	6.92	4
FASAA	9	2355‐31‐9	N‐MeFOSAA	3.06	8.13	31.3	4
	12	2991‐50‐6	N‐EtFOSAA	<1.0	2.70	11.5	4
FTCA	8	914637‐49‐3	5:3 FTCA	5.74	7.76	<2.0	0
FTS	8	27619‐97‐2	6:2 FTS	<1.0	0.621	<1.0	0
	10	39108‐34‐4	8:2 FTS	<0.5	0.608	1.32	0
			ƩPFAS	51.1	371	185	

Abbreviations: CAS, Chemical Abstracts Service Registry Number; FASA, perfluoroalkyl sulfonamides; FASAA, perfluoroalkyl sulfonamido acetic acids; FTCA, fluorotelomer carboxylic acid; FTS, fluorotelomer sulfonate; N‐EtFOSAA, N‐ethyl perfluorooctane sulfonamidoacetic acid; N‐MeFOSAA, N‐methyl perfluorooctane sulfonamidoacetic acid; PFBA, perfluorobutanoic acid; PFBS, perfluorobutane sulfonate; PFCA, perfluoroalkyl carboxylate; PFDA, perfluorodecanoic acid; PFDoA, perfluorododecanoic acid; PFDS, perfluorodecane sulfonate; PFHpA, perfluoroheptanoic acid; PFHpS, perfluoroheptane sulfonate; PFHxA, perfluorohexanoic acid; PFHxS, perfluorohexane sulfonate; PFNA, perfluorononanoic acid; PFOA, perfluorooctanoic acid; PFOS, perfluorooctane sulfonate; PFOSA, perfluorooctane sulfonamide; PFPeA, perfluoropentanoic acid; PFPeS, perfluoropentane sulfonate; PFSA, perfluoroalkyl sulfonate; PFTeDA, perfluorotetradecanoic acid; PFTrDA, perfluorotridecanoic acid; PFUnA, perfluoroundecanoic acid.

A review by Behnami et al. (2024) summarized PFAS concentrations in biosolids from numerous studies around the world. Biosolids in the United States showed a range of ƩPFAS concentrations, from <MDL to 1017 ng g^−1^_,_ although it should be noted that each study in the review defined ƩPFAS differently. Even so, ƩPFAS results presented in this study are consistent with concentrations reported by Behnami et al. (2024) in US biosolids. A recent study by Oviedo‐Vargas et al. (2025) measured PFAS in biosolids and soils at working farms where the biosolids were applied. ƩPFAS in biosolids (144–350 ng g^−1^) were similar to concentrations found in this study. Recent studies have determined that precursors and intermediates that can degrade to perfluoroalkyl acids make up the bulk of PFAS present in biosolids (Alvarez‐Ruiz et al., 2024; Schaefer et al., 2022). However, commonly used analytical methods including methods used in this study, quantify only a few precursors and therefore likely underestimate actual total PFAS concentration.

In 2025, US EPA released a draft risk assessment (US EPA, 2025), which evaluated the risk associated with exposure to perfluorooctanoic acid (PFOA) and PFOS in land‐applied biosolids in specific scenarios. The draft risk assessment found that land application of biosolids containing 1 part per billion (ng g^−1^) of PFOA or PFOS may pose a risk to those living on or near application sites, including farm families and their neighbors. The risk calculations in the EPA assessment are not considered conservative (protective) because they used median exposure conditions rather than the usual 95th percentile. PFOS at all three facilities tested in this study exceeded 1 ng g^−1^, while PFOA was >1 ng g^−1^ at facilities H and I.

Also in 2025, Minnesota Pollution Control Agency (MPCA) began implementing its Minnesota Biosolids PFAS Strategy (hereafter referred to as the “Strategy”), as directed by the Minnesota State Legislature (MPCA, 2025). The Strategy requires all WWTPs that intend to apply their biosolids to agricultural land or reclamation projects to test for PFAS using EPA Method 1633. PFOS and PFOA results are evaluated using tiered thresholds, with each concentration threshold requiring specified actions. For example, PFOS and PFOA concentrations in biosolids B and H fall into Tier 1 (PFOS or PFOA ≤ 20 ng g^−1^) which requires the WWTP to notify the landowner and farmer that results are available. PFOS was present at 49.5 ng g^−1^ in biosolid I, which is near the upper limit of Tier 2 (21–50 ng g^−1^), which requires notification to the farmer and landowner, creation and implementation of a pollutant minimization plan, and reporting to MPCA.

### 3.2 Soil

**FIGURE 1:**
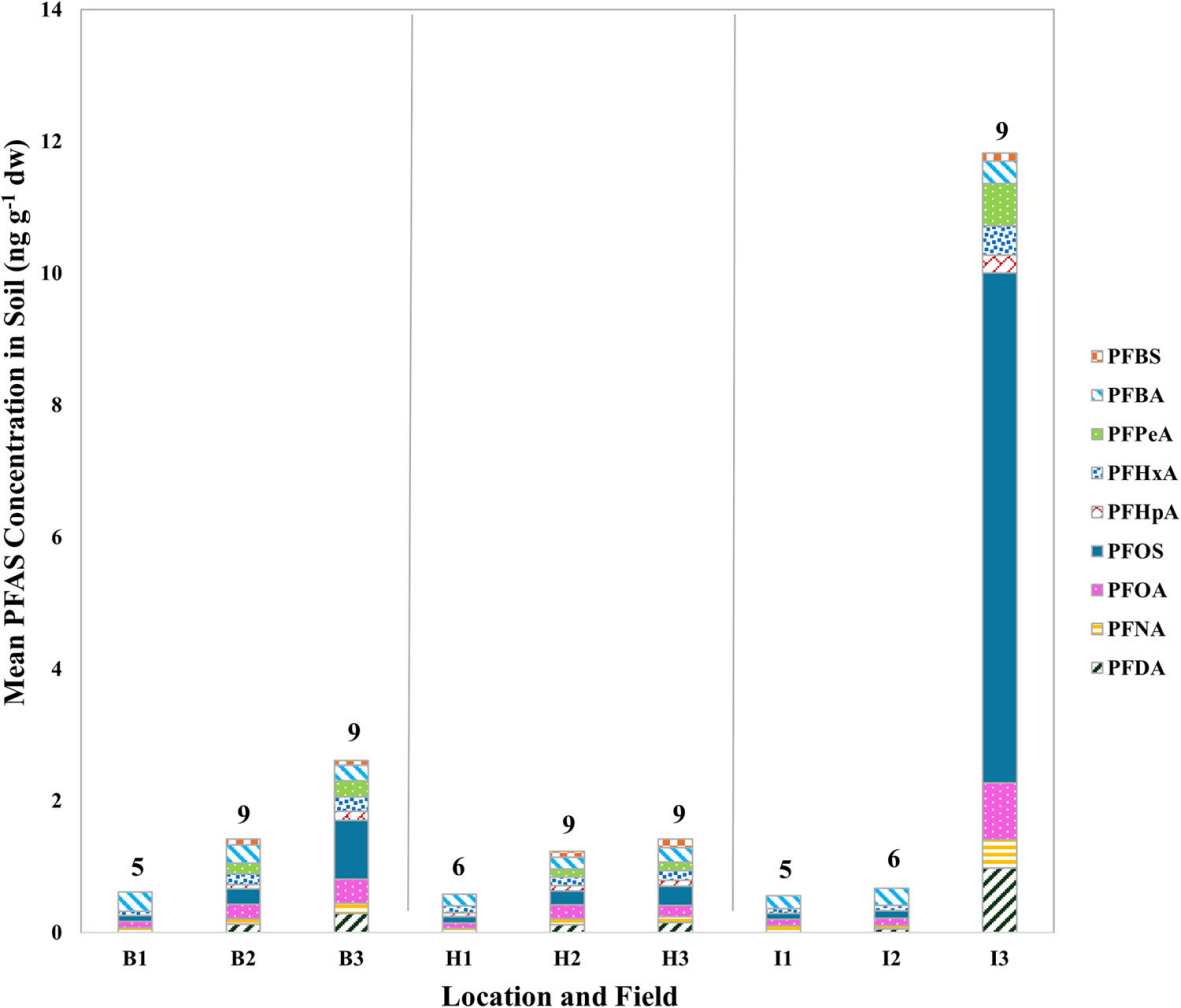
Mean ƩPFAS concentration for the nine focal PFAS in all soil samples at each location and field. Results from all depths and sampling dates were used to calculate mean concentrations. The number above each column is the number of PFAS detected at that field. Field 1 at all locations had no history of biosolids application. Field 2 at all locations had biosolids applied *at least* 2 years prior to this study. Field 3 received biosolids during the study year. All field 3 locations had the highest mean ƩPFAS concentration, demonstrating the impact of biosolids application on soil. PFAS, per‐ and polyfluoroalkyl substances; PFBA, perfluorobutanoic acid; PFBS, perfluorobutane sulfonate; PFDA, perfluorodecanoic acid; PFHpA, perfluoroheptanoic acid; PFHxA, perfluorohexanoic acid; PFNA, perfluorononanoic acid; PFOA, perfluorooctanoic acid; PFOS, perfluorooctane sulfonate; PFPeA, perfluoropentanoic acid.

A total of 21 PFAS were detected at least once across all soil samples from all three locations, but only 19 PFAS were present in more than one soil sample (Table 1; Figure S4). Nine PFAS were present in ≥15% of all soil samples (Table 1) and were considered “frequently detected.” These nine PFAS are the focus of analysis in this study (Figure 1). Relative detection frequencies of the nine focal PFAS in all soil samples were PFOA > perfluorobutanoic acid (PFBA) > PFOS > PFBS > perfluorohexanoic acid > perfluoroheptanoic acid > PFNA > perfluorodecanoic acid > perfluoropentanoic acid (PFPeA). The complete dataset is presented in Table S5. The detection frequency of each PFAS in soil was higher in the fields where biosolids were applied compared to the fields with no history of application (Table S5). For example, PFOS was found in 52% and 55% of samples at location B, field 1 and field 2, respectively, compared to 86% of samples at location B, field 3. This pattern is even more pronounced at location I, where PFOS was found in 22%, 33%, and 100% of soil samples from fields 1, 2, and 3, respectively.

Four of the nine focal PFAS had maximum concentrations <1 ng g^−1^ dw in soil. PFOS had the highest maximum concentration in soil at location I, field 3 (18.0 ng g^−1^ dw) (Table S5), a result which aligned well with PFOS concentration in biosolid I. The overall mean PFOS concentration (all fields, sampling dates, and depths, combined) was 1.76 ± 3.56 ng g^−1^ dw. However, when only field 3 soil samples were considered, the mean PFOS concentration (all locations, sampling dates, and depths, combined) was 2.94 ng g^−1^ dw, which is similar to the median PFOS concentration (2.7 ng g^−1^) of 5700 soil samples from >1400 sampling locations in six nations, but much lower than the maximum PFOS concentration in soils where biosolids were applied (0.4–1409 ng g^−1^) (Brusseau et al., 2020)

The number of PFAS detected in soil increased in fields treated with biosolids compared to those with no history of application (Figure 1; Figure S4). The nine focal PFAS made up the majority of the ƩPFAS (80%–100%) at all locations. Indeed, with the exception of locations B3 and I3, there was no significant difference between the sum of the mean concentration of the nine focal PFAS and the sum of the mean concentration of all detected PFAS for each site (Figure 1; Figure S4).

Based on the results shown in Figure 1, it is clear that the application of biosolids impacts both the number of PFAS present in soil as well as the concentration. Compared to biosolids‐treated sites in recent studies, field 3 soil samples generally had similar or lower PFAS concentrations. For example, in a study on biosolids‐treated farms in the northeastern United States, median ƩPFAS (sum of 0–15 and 15–30 cm) was 29.3 ng g^−1^ (Oviedo‐Vargas et al., 2025), compared to median ƩPFAS (0–30 cm) of 2.47, 1.39, and 13.4 ng g^−1^ in field 3 of locations B, H, and I, respectively. An agricultural site with a long history of biosolids application had much higher ƩPFAS up to 133.4 ng g^−1^ in the upper vadose zone soil, even with a 6‐year break in application (L. G. Peter, Lee, et al., 2025). This is likely due to repeated biosolids application, which can enhance retention of PFAS in surficial soil, as demonstrated by Garza‐Rubalcava et al. (2026).

Fields with no record of biosolids application had only a few PFAS present at concentrations on par with currently understood “ambient background” soil concentrations in Minnesota and around the world. For example, Rankin et al. (2016) reported PFOS concentrations (0.304 and 0.112 ng g^−1^) in two Minnesota ambient background soil samples that are comparable to or slightly higher than the mean PFOS concentration measured at field 1 in locations B (0.06 ng g^−1^), H (0.10 ng g^−1^), and I (0.10 ng g^−1^). The same study tested soil from Antarctica and found similar, low PFAS concentrations (0.19 ng g^−1^, ƩPFAS) (Rankin et al., 2016).

**FIGURE 2:**
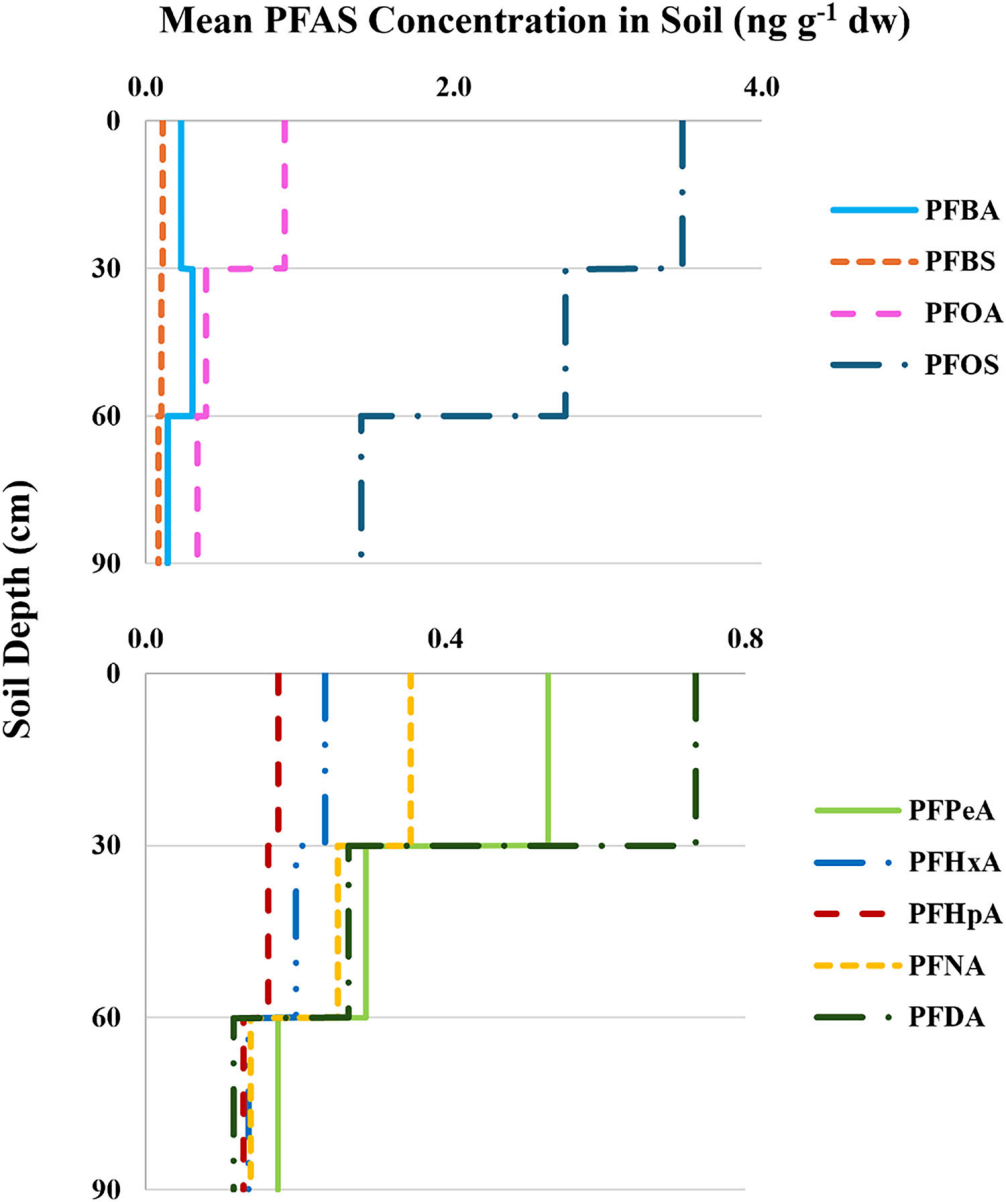
Change in mean per‐ and polyfluoroalkyl substances (PFAS) concentration with depth in the soil profile (0–90 cm) at all field 3 locations. Results from all locations and sampling dates were used to calculate the mean concentration. The upper chart shows a four‐carbon carboxylate (perfluorobutanoic acid [PFBA]) and sulfonate (perfluorobutane sulfonate [PFBS]), and an eight‐carbon carboxylate (perfluorooctanoic acid [PFOA]) and sulfonate (perfluorooctane sulfonate [PFOS]). The lower chart shows carboxylates with carbon chain length ranging from 5 to 10. In general, long‐chain PFAS and sulfonates are retained in soil compared to more mobile short‐chain PFAS and carboxylates. PFDA, perfluorodecanoic acid; PFHpA, perfluoroheptanoic acid; PFHxA, perfluorohexanoic acid; PFNA, perfluorononanoic acid; PFPeA, perfluoropentanoic acid.

An examination of individual PFAS concentrations at depth in the soil column indicates that, in general, the highest concentration of each PFAS occurred in the surficial (0–30 cm) soil layer (Figure 2; Figures S5 and S6). This is particularly true for the longer chain, relatively less‐mobile PFAS like PFOS. Conversely, the concentration of PFBA, a short‐chain carboxylate, appears to slightly increase in the 30‐ 60‐cm layer, pointing to greater mobility in the soil column. These results are consistent with results presented in a recent study evaluating leaching of PFAS from biosolids‐amended soils in saturated flow‐through columns (Manzur et al., 2025). In that study, up to 98% of PFAS were retained in post‐leaching soils and observed PFAS mobility through the soil column was chain‐length dependent. In addition, the study by Manzur et al. (2025) found that the same transport parameters could be used to describe PFAS mobility in biosolids‐amended soil, despite the application of biosolids with different properties, suggesting that background soils and water flow regimes will control PFAS transport.

Several factors can impact PFAS mobility in soil, including the physicochemical properties of individual PFAS, soil properties, and water flow regimes. Chain length and functional group govern PFAS water solubility and soil mobility, where shorter chain length PFAS (*C* ≤ 6) are generally more water soluble and mobile than longer chain PFAS (*C* > 6) (Brusseau & Guo, 2022). In general, perfluoroalkyl carboxylates (PFCAs) are more water soluble and more mobile in soils compared to perfluoroalkyl sulfonates (PFSAs) with the same chain length (Cai et al., 2022). Soils properties such as lower pH, higher organic carbon content, and higher silt and clay content tend to increase PFAS sorption to soil, leading to reduced bioavailability (Campos‐Pereira et al., 2023). In addition, precursor transformation in the field can lead to the formation of more‐mobile short‐chain PFAS, resulting in greater migration through the soil (Garza‐Rubalcava et al., 2026; Gebbink et al., 2015; Schaefer et al., 2022).

While changes in PFAS concentration over the growing season appear, based on visual inspection, to slightly decrease for most PFAS in soil from preplanting to postharvest (Figures S7 and S8), this decrease was not significant in a paired *t*‐test (*p* > 0.05). This apparent decrease is likely the result of a combination of spatial heterogeneity in contaminant concentration and analytical variability rather than downward migration through the soil profile or uptake into plants. Of note, this growing season experienced lower‐than‐average precipitation, which may have reduced downward leaching, overland flow, and plant uptake. A literature search at the time of writing did not locate any studies detailing changes in PFAS soil concentration over the course of a single growing season. A more detailed seasonal mass balance study would be helpful in elucidating short‐term movement of PFAS in agricultural soils.

### 3.3 Plants

Up to five PFAS, including PFMPA (perfluoro‐3‐methoxypropanoic acid), PFBS, PFBA, PFOS, and PFPeA were detected more than once in a set of triplicate crop samples (Table S5). Although PFMPA, a perfluoroalkyl ether carboxylate, was not detected in any biosolids or soil samples, it was present in corn stover at all three locations, with the highest concentration present in stover grown at location B, field 3 (1.0 ng g^−1^; Table S4). It is possible that PFMPA was present in biosolids and/or soils at concentrations below the MDL, becoming detectable only after accumulating in plant tissue. It is also possible that the source of the PFMPA in corn stover was something other than biosolids (e.g., pesticide inert ingredient), or even some other local source. Given the anonymity of the farm locations, it is not possible to determine whether any potential local sources of PFMPA were present near any of the participating farms. The fate of perfluoroalkyl ether acid precursors in water was explored by C. Zhang et al. (2019) using the total oxidizable precursor (TOP) assay. In that study, ADONA (4,8‐dioxa‐3H‐perfluorononanoic acid) was completely transformed into PFMPA. Likewise, Cioni et al. (2022) demonstrated that 100% of ADONA in human serum could be transformed into PFMPA using a modified TOP assay. It is possible that PFMPA was present as a result of precursor transformation, although ADONA was not detected in any biosolids or soil samples in this study, so this cannot be verified. Regardless, PFMPA BAFs could not be calculated due to a lack of detections in soil. However, a study by W. Zhang et al. (2021), demonstrated plant uptake of five ether‐PFAS, including PFMPA, which accumulated in plant roots and shoots. The same study also examined mass fractionation of ether‐PFAS in soil and found that the majority of PFMPA was present in the water‐soluble fraction, indicating PFMPA in porewater would likely be bioavailable to plants. Future studies of plant uptake in field settings would be improved by analyzing porewater in addition to soil.

PFBA and PFBS were detected in corn stover at all three locations at mean concentrations up to 2.4 and 0.60 ng g^−1^ dw, respectively (Table S4). The maximum concentrations for both PFBA and PFBS occurred at location I, field 3. PFOS was found in corn stover only at location I, field 3, which was the location that received biosolids with the highest PFOS concentration (Table 1). Location I, field 3 also had the highest PFOS soil concentration (Figure 1; Table S5). The mean concentration of PFOS in corn stover was 0.21 ng g^−1^ dw (Table S4). A study by Krippner et al. (2015) demonstrated PFAS accumulated in corn stover but not kernels when grown on soil spiked with concentrations of PFAS much higher than in this study. Several studies examining PFAS uptake in plants have demonstrated that PFAS transfer to reproductive and storage organs is low relative to transfer to stalks and leaves (Blaine et al., 2014; Lechner & Knapp, 2011; Stahl et al., 2009). In general, bioaccumulation of PFAS is higher in plant stems and leaves than reproductive and storage organs, and bioaccumulation in aboveground plant parts decreases with increasing PFAS chain length (Lesmeister et al., 2021). Overall, relative PFAS transfer from soil to plants is roots > leaves > fruits (EFSA CONTAM Panel, 2020).

Low levels of PFBA (0.16 ng g^−1^ dw, maximum) were also detected in rye grown at location B, field 1, which did not have a history of biosolids application but did have measurable PFBA in soil (0.33 ng g^−1^ dw, maximum), which may be attributable to atmospheric deposition (Table S4). PFBA is commonly detected in air and precipitation, even in remote areas (Ahrens et al., 2023; Dreyer et al., 2009). In a year‐long study of PFAS in air at multiple locations in Minnesota, PFBA was both the most frequently detected PFAS (100% of samples) and the most abundant PFAS in terms of concentration (MPCA, 2022). In addition, four conventional fertilizers tested prior to the start of this study did not have any detectable PFAS, making conventional fertilizers an unlikely source of PFAS to fields that did not receive biosolids (Table S5).

No PFAS were detected in any of the soybean samples, but that should not be taken as an indication that soybeans cannot accumulate PFAS. Several studies have demonstrated accumulation of ƩPFAS in soybeans as high as 8085 ng g^−1^ (Jiang et al., 2022; X. Li et al., 2024; Liu et al., 2019; W. Zhang et al., 2022), primarily at sites irrigated with highly contaminated water (Omagamre et al., 2025) or grown in fields with highly contaminated soils (Liu et al., 2019). In contrast, soybeans in this study were rainfed and grown in fields that either had no history of biosolids application or application at least2 years prior to the start of the study with low ƩPFAS in soils. These findings indicate that PFAS may have limited transfer to soybean grain under the conditions evaluated in this study.

Soil‐plant BAFs were derived for PFBS (5.0–5.2, stover), PFBA (7.0–7.7, stover; 0.57, rye), PFPeA (0.34, stover), and PFOS (0.03, stover) (Table S7). BAFs calculated in this study were comparable to BAFs presented in a review of >4500 soil–plant BAFs in agricultural plants (Lesmeister et al., 2021). The BAF for PFBA in rye was about one order of magnitude lower than in corn stover, perhaps reflecting a difference in the uptake capacity of rye compared to corn given similar soil PFBA concentrations (0.28 ng g^−1^ dw in rye field, 0.18–0.34 ng g^−1^ dw in corn fields; Table S4). PFOS, although present in corn stover, had the lowest BAF indicating a tendency for PFOS to remain in soil under conditions present in this study. Furthermore, a BAF could only be calculated for PFOS for corn stover harvested from location I, field 3, which received biosolids with the highest PFOS concentration in this study, perhaps indicating a minimum concentration of PFOS in soil is required before uptake can occur. The PFOS BAF in corn stover in this study is lower than the median PFOS BAF in corn stover (0.17) calculated in a field study by Bhattacharya et al. (2025); however, the maximum PFOS soil concentration in that study (190 ng g^−1^ dw) was an order‐of‐magnitude higher than the maximum soil concentration in this study (18 ng g^−1^ dw).

Plant uptake of PFAS is governed by a number of factors, including the physicochemical properties of individual PFAS (e.g., carbon chain length and functional group), soil properties (e.g., soil texture, organic matter content, pH, and mineral content), and plant tissue type (e.g., root, shoot, and fruit) (Bolan et al., 2021; Ghisi et al., 2019; Lesmeister et al., 2021; J. Li et al., 2022; Simones et al., 2024). PFAS BAFs in shoots, leaves, and fruits (reproductive organs) decreased (i.e., less plant uptake) with increasing PFAS carbon‐chain length in a linear relationship (Lesmeister et al., 2021), indicating that short‐chain PFAS (*C* ≤ 6) are more likely to accumulate in the aerial parts of plants than long‐chain PFAS (*C* > 6). Conversely, BAFs in plant roots increased with increasing chain length in either a linear or U‐shaped relationship (Lesmeister et al., 2021), indicating that long‐chain PFAS like PFOS are more likely to be present in plant roots. This may be related, in part, to water solubility of individual PFAS. Water‐soluble PFCAs are expected to have higher porewater concentrations and thus a higher bioavailable fraction compared to less‐water soluble PFSAs of the same chain length, resulting in a greater potential for plant uptake (W. Zhang et al., 2021). Under some conditions, PFAS in shallow porewater following biosolids application will be the primary concern for plant uptake, as described by Manzur et al. (2025). A possible limitation of this study is a lack of porewater data for use in BAF derivation, which may provide a more accurate estimate of plant uptake.

### 3.4 Potential dietary risk

Dietary exposure can be estimated by combining food consumption data with chemical concentration data. When chemical concentration data for certain foods are not readily available, the concentrations can be estimated with BTFs if sufficient information is available (Leeman et al., 2007). Dietary risk assessment should consider exposure of sensitive subpopulations (e.g., children, women of childbearing age, subsistence populations, etc.) as they may have higher exposure (e.g., consumption rates) and/or be more sensitive to the effects of the chemical than the average consumer. Risk estimates can then be developed using data about the exposure of the relevant population or subpopulation and the chemical's toxicity.

Soil and crop data from this study were used to estimate potential risk to human consumers of beef and milk produced by hypothetical cattle fed, in part, on the corn stover grown at location I, field 3. The results of the risk calculations are presented in Table 2. Details of the risk calculations are provided in Figures S2 and S3, and Table S6. The HQs for total ingestion (beef + milk) for both adults (1.1) and children (6.9) were >1 based on the 90th percentile consumption rates, while only the child HQ was >1 using mean consumption rates. If the HQ is ≤1, then the risk is estimated to be acceptable. If the HQ is >1, potential for risk may be present and the situation should be investigated further. The risk driver in these calculations was cattle consuming contaminated corn silage, represented by the mean PFOS concentration in corn stover grown at location I, field 3. While the assessment is based on a hypothetical scenario, a similar real‐world situation would warrant additional investigation, which could include additional sample collection and/or testing the actual animal products to determine whether calculated screening levels in beef and milk are exceeded.

**TABLE 2:** Estimate of risk to consumers of beef and milk.

		Consumption rate (g kg^−1^ bw day^−1^)		PFOS screening value(ng kg^−1^)		Hazard quotients (HQs)	
Animal product	Population (age)	Mean	90th percentile	Mean	90th percentile	Mean	90th percentile
Beef^a^	Child (1 to <6)	2.3	4.7	348	170	0.47	1.0
Milk^b^	Child (1 to <6)	30.9	58.9	26	14	3.1	6.0
				Total ingestion child		3.6	6.9
Beef^a^	Adult (20+)	1.1	2.2	727	364	0.22	0.45
Milk^b^	Adult (20+)	2.7	6.1	296	131	0.27	0.62
				Total ingestion adult		0.5	1.1

Abbreviation: PFOS, perfluorooctane sulfonate.

^a^ PFOS concentration in beef muscle = 162 ng kg−1.

^b^ PFOS concentration in milk = 81 ng kg−1.

#### 3.4.1 Uncertainty analysis

Uncertainties are present in the risk assessment process because risk calculations are typically based on a number of assumed conditions. While not an exhaustive list, the following core assumptions introduce uncertainty and may therefore under‐ or overestimate risk:

1. The calculations are based on a limited number of actual soil and plant sample data. This may under‐ or overestimate risk.
2. The calculations assume that the majority of PFOS exposure for cattle is from contaminated corn stover (assumed to represent corn silage). This assumption may underestimate risk if the cattle are also exposed to contaminated water and/or soil, or it may under or overestimate risk if the cow's diet composition is different. Additionally, this assessment assumes corn silage is entirely composed of corn stover—this assumption likely overestimates risk. Silage is typically composed of both stover and ears. As shown in Krippner et al. (2015), PFAS tend to accumulate in stover but not in kernels of corn grown on soil spiked with PFAS. PFOS was also not detected in corn ear samples in this study.
3. The calculations assume that the child's or adult's total beef and milk consumption comes from impacted animals. This assumption likely overestimates risk.
4. The beef and milk BTFs used in the calculations are derived from a single study—this may under or overestimate risk.

Because of the uncertainties summarized above, none of the risk calculations presented here should be interpreted as precise measures of true risk. Rather, all values should be interpreted as uncertain estimates.

## 4 CONCLUSIONS

While numerous studies have demonstrated that PFAS in biosolids pose a potential risk to human and environmental health, relatively few studies have evaluated PFAS in soils over the course of a growing season and plant uptake at multiple farms and fields with a range of biosolid application histories. Biosolids with higher concentrations and a greater number of PFAS have greater impact on amended soils, increasing the likelihood that crops grown in those soils could also be contaminated. Evaluation of PFAS uptake in crops grown in fields under typical US agricultural conditions (i.e., not highly contaminated sites) are scarce, so data presented here are important validation of the outcomes of prior laboratory‐ and greenhouse‐based studies. Testing multiple fields with a range of biosolids application histories within each farm is unique, and results presented here demonstrate clear impacts of biosolids application on PFAS soil concentration and plant uptake. Furthermore, the risk assessment modeling presented here demonstrate the potential for risk to consumers of beef and milk when cows are fed a diet that includes corn stover with relatively low (<1 ng g^−1^) PFOS concentrations. Addressing potential risks from land‐applying PFAS‐contaminated biosolids will require a multipronged approach focused on source reduction.

## AUTHOR CONTRIBUTIONS

**Summer Streets**: Conceptualization; formal analysis; funding acquisition; methodology; project administration; supervision; visualization; writing—original draft; writing—review and editing. **Emerson F. C. Souza**: Formal analysis; writing—original draft; writing—review and editing. **Matthew McNearney**: Investigation; methodology; resources. **Sona Jedinak**: Formal analysis; writing—original draft; writing—review and editing. **Alonso Doria Manzur**: Investigation; resources; writing—review and editing. **Jennifer L. Guelfo**: Methodology; resources; writing—review and editing. **Carl Rosen**: Conceptualization; methodology; project administration; supervision; writing—review and editing.

## CONFLICT OF INTEREST STATEMENT

The authors declare no conflicts of interest.

## Supporting information



Supplemental materials include figures and tables that provide additional data exposition and interpretation. The complete dataset, including QA/QC data are provided. Methods descriptions with additional analytical and human‐health risk assessment details are also provided.

## Data Availability

The datasets generated during and/or analyzed during the current study are available in the MPCA File Server repository at https://files.pca.state.mn.us/pub/file_requests/datasets/PFAS/Table_S5_PFAS_in_Land‐Applied_Biosolids_Streets.xlsx.
