# Supplementary material for: Per‐ and polyfluoroalkyl substances in land‐applied biosolids: Accumulation in soils, crop uptake, and potential dietary risk
Source: J Environ Qual. 2026 Jul 15;55(4):e70220. doi: 10.1002/jeq2.70220 (PMC13373352; doi:10.1002/jeq2.70220)
Supplement: Supplementary file 1 — Supplemental materials include figures and tables that provide additional data exposition and interpretation. The complete dataset, including QA/QC data are provided. Methods descriptions with additional analytical and human‐health risk assessment details are also provided. [file JEQ2-55-0-s001.docx]

**PFAS in land-applied biosolids: Accumulation in soils, crop uptake, and potential dietary risk**

Summer Streets, Emerson F.C. Souza, Matthew McNearney, Sona Jedinak, Alonso Doria Manzur, Jenn Guelfo, Carl Rosen

**30 pages**

**Supplemental Information**


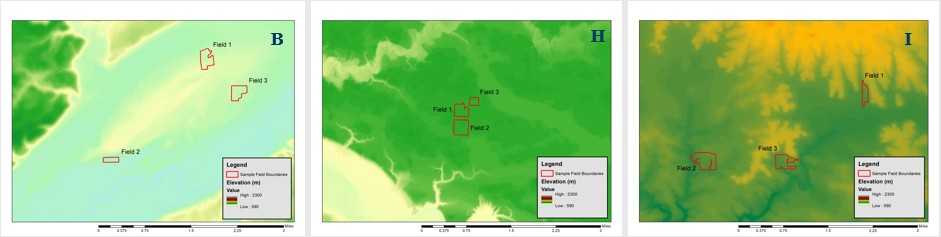


**Supplemental Figure S1**. Elevations, relative areas, and distance between fields at locations B, H, and I.

*Application rates and treatment types*

Facility B used aerobic treatment and provided Class A biosolids that were de-watered and heat dried. Facility B biosolids were applied to location B, field 3 (sandy loam soil) in spring of 2023 at an estimated rate of 12.4 Mg ha^-1^ (dry source). Facility H used aerobic treatment and provided Class A liquid biosolids that were applied by injection at a depth of 20 – 30 cm in fall of 2022. Facility H biosolids were applied to location H, field 3 (clay loam soil) at an estimated rate of 4.27 Mg ha^-1^ (liquid source). Facility I used anaerobic treatment and provided Class B liquid biosolids that were applied by injection at a depth of 20 – 30 cm in fall of 2022. Facility I biosolids were applied to location I, field 3 (silt loam soil) at a rate of 5.21 Mg ha^-1^ based on applicator records. Sample handling precautions were followed at all times, as described in the section on sampling precautions in this manuscript.

*Sampling precautions*

Prior to soil sample collection, all equipment (e.g. probes, buckets, shovels, sampling tubes) was washed with Alconox detergent, rinsed with ultra-pure deionized water three times, rinsed with methanol one time, followed by a final rinse with ultra-pure deionized water that was collected and analyzed for PFAS (equipment rinse blank). Crops were hand harvested at three sites within each field near the areas where soil samples were collected based on GPS coordinates. Knives with stainless steel blades were used to cut plants in the field. Knives were pre-cleaned in the lab following the same cleaning procedure used on other field equipment. In the field, knives were cleaned again with the same 3-step procedure used in the lab and rinsed with laboratory-grade PFAS-free water prior to the first cut and in between each sample.

Precautions to avoid PFAS contamination of samples as detailed in Michigan Department of Environmental Quality (DEQ) Fish Tissue PFAS Sampling Guidance (EGLE 2024), the quality assurance project plan (QAPP) developed by the Minnesota Pollution Control Agency (MPCA, available upon request), and laboratory guidance were followed at all times during sampling and handling. All equipment was thoroughly cleaned and rinsed as described above, and the rinsate was analyzed for PFAS. Low concentrations (0.56 – 5.3 ng/L, parts per trillion) of certain PFAS congeners were present in the rinse blanks of some blank samples (Table S2). However, concentrations were 1 – 2 orders of magnitude lower than method detection limits for soil and vegetation, therefore it is unlikely that the trace levels of PFAS present in some of the rinse blanks had a significant impact on soil and vegetation results. Nonetheless, PFAS detections in the equipment blanks were checked against corresponding sample data, and sample concentrations < 10x the equipment blank concentration were rejected.

|  |  | **Supplemental Table S1.** Number of samples collected, timing of sample collection, and analyses performed. | | | | | | | | | |
| --- | --- | --- | --- | --- | --- | --- | --- | --- | --- | --- | --- |
|  |  | **Sample Type** | | | | | | | | | |
|  |  | Soil | | | | | | Crop | | | |
| **Location & Field** |  | Depth (cm) | pre-planting | early season | mid-season | post-harvest | n (total) | Analyses | Type | n | Analyses |
|  | B1 | 0-30 | 3 | 3 | 3 | 3 | 12 | EPA 1633 UMRAL | Rye | 3 | EPA method 1633 |
|  |  | 30-60 | 3 | 3 | 3 | 3 | 12 |  |  |  |  |
|  |  | 60-90 | 3 | 3 | 0 | 3 | 9 |  |  |  |  |
|  | B2 | 0-30 | 3 | 3 | 3 | 3 | 12 | EPA 1633 UMRAL | Soybean | 3 | EPA 1633 |
|  |  | 30-60 | 3 | 3 | 3 | 3 | 12 |  |  |  |  |
|  |  | 60-90 | 3 | 3 | 0 | 3 | 9 |  |  |  |  |
|  | B3 | 0-30 | 3 | 3 | 3 | 3 | 12 | EPA 1633 UMRAL | Corn, Ears & Stover  (3 each) | 6 | EPA 1633 |
|  |  | 30-60 | 3 | 3 | 3 | 3 | 12 |  |  |  |  |
|  |  | 60-90 | 3 | 3 | 0 | 3 | 9 |  |  |  |  |
|  | H1 | 0-30 | 3 | 3 | 3 | 3 | 12 | EPA 1633 UMRAL | Corn, Ears & Stover  (3 each) | 6 | EPA 1633 |
|  |  | 30-60 | 3 | 3 | 3 | 3 | 12 |  |  |  |  |
|  |  | 60-90 | 3 | 3 | 0 | 3 | 9 |  |  |  |  |
|  | H2 | 0-30 | 3 | 3 | 3 | 3 | 12 | EPA 1633 UMRAL | Soybean | 3 | EPA 1633 |
|  |  | 30-60 | 3 | 3 | 3 | 3 | 12 |  |  |  |  |
|  |  | 60-90 | 3 | 3 | 0 | 3 | 9 |  |  |  |  |
|  | H3 | 0-30 | 3 | 3 | 3 | 3 | 12 | EPA 1633 UMRAL | Corn, Ears & Stover  (3 each) | 6 | EPA 1633 |
|  |  | 30-60 | 3 | 3 | 3 | 3 | 12 |  |  |  |  |
|  |  | 60-90 | 3 | 3 | 0 | 3 | 9 |  |  |  |  |
|  | I1 | 0-30 | 3 | 3 | 3 | 3 | 12 | EPA 1633 UMRAL | Soybean | 3 | EPA 1633 |
|  |  | 30-60 | 3 | 3 | 3 | 3 | 12 |  |  |  |  |
|  |  | 60-90 | 3 | 3 | 0 | 3 | 9 |  |  |  |  |
|  | I2 | 0-30 | 3 | 3 | 3 | 3 | 12 | EPA 1633 UMRAL | Soybean | 3 | EPA 1633 |
|  |  | 30-60 | 3 | 3 | 3 | 3 | 12 |  |  |  |  |
|  |  | 60-90 | 3 | 3 | 0 | 3 | 9 |  |  |  |  |
|  | I3 | 0-30 | 3 | 3 | 3 | 3 | 12 | EPA 1633 UMRAL | Corn, Ears & Stover  (3 each) | 6 | EPA 1633 |
|  |  | 30-60 | 3 | 3 | 3 | 3 | 12 |  |  |  |  |
|  |  | 60-90 | 3 | 3 | 0 | 3 | 9 |  |  |  |  |
|  |  |  |  |  |  | **Total Soil Samples** | 297 |  | **Total Crop Samples** | 39 |  |
|  |  |  |  |  |  |  |  |  |  |  |  |

*PFAS analysis (Texas Tech University)*

Biosolid extraction for targeted analysis was performed using a previously published, basic methanol extraction (Guelfo and Higgins, 2013; Nickerson et al., 2021; Sepulvado et al., 2011). Briefly, 0.2-0.5 g (dry weight) of homogenized biosolids were weighed into 50 mL polypropylene centrifuge tubes and spiked with 4 ng of each IS. A 7 mL aliquot of basic methanol (1% [V/V] ammonium hydroxide in Liquid chromatography-mass spectrometry , LC-MS, grade methanol) was added to each tube and vortexed for 30 s. Samples were then added in a heated sonication bath for 1 hr, and placed on a shaker table (VWR, 3500STD) for 2 hr. Samples were then centrifuged for 30 min at 3180 xg (centrifuge Eppendorf 5810) and supernatant was transferred into a clean 20 mL glass vial. After 3 rounds of extractions, combined extracts were evaporated under nitrogen and reconstituted in 1000 µL acidic methanol (1% [V/V] acetic acid in LC-MS grade methanol). Reconstituted samples were transferred to a microcentrifuge tube containing 20 - 40 mg of dispersed ENVI-carb, vortexed for 30 s, and centrifuged (20627 xg for 30-min, Beckman Coulter Microfuge® 20 Centrifuge). For targeted analysis, 126 μL of cleaned, methanol extracts were diluted (14x) with LC-MS grade methanol and ultrapure, deionized water (DI) to achieve a final autosampler vial composition of 50% water and 50% methanol containing 200 ng/L of each IS.

*Instrumental data acquisition, processing, and quality control*

Chromatographic separation was performed on a C18 analytical column (Gemini®, 3 uM, 100 X 3 mm ID, Phenomenex, CA, USA) coupled with a guard column (Gemini®, C18 4 x 2.0mm ID, Phenomenex, CA, USA) with a SCIEX Exion LC high pressure liquid chromatographer (HPLC). A delay column (Luna®, 5 µm, C18, 30 x 3mm, Phenomenex, CA, USA) was installed between the mobile phase mixer and sample injection to minimize background contamination from solvent reservoir tubing and pump parts. Mobile phase consisted of an aqueous and organic phase. The aqueous solution (A) consisted of a 2 mM ammonium acetate solution in ultrapure water. The organic phase (B) was 100% LC-MS grade methanol. Mobile phase flow rate was maintained at 600 μL/min throughout the run, while it composition was ramped from 95% A to 35% A over the first minute, 5% A at 8 minutes, 1% A at 8.1 minutes and then kept constant for 12.5 minutes until the end of the run where composition was ramped to 95% A (13 minutes) and equilibrated the column for 3.5 minutes.

All analytic acquisitions were performed on a quadrupole time of flight-mass spectrometry (QTOF-MS) system (X500R, SCIEX, Framingham, MA, USA). Turbo ion spray was used as the ion source and maintained at 500⁰C during the samples acquisition with the following conditions: Ion spray voltage -4500 (v); curtain gas 30 (PSI); ion source gas 1 40 PSI, ion source gas 2 60 PSI. Collision activated dissociation (CAD) gas was maintained at 10 PSI. Ultra-pure nitrogen was used for source, exhaust, and CAD gases. The HPLC-QTOF-MS was operated in a Multiple reaction monitoring high resolution (MRM-HR) mode. 52 PFAS (Table S4) were monitored using a MRM-HR acquisition method that acquired two transitions (quantifier and qualifier) for each PFAS, where possible (Table S5). besides data were acquired and processed using SCIEX OS (versions 2.2). PFAS were quantified using isotope dilution over a calibration range of 0.5-5000 ng/L (R2>0.99).

HPLC-QTOF-MS was used in full-scan mode to collect MS data. MS/MS spectra were acquired for 40-2500 Da range using a sequential window acquisition of all theoretical mass spectra (SWATH) mode, which is a data independent acquisition method. The MS scans (50-2500 Da) were divided into 10 equally spaced windows (e.g., 50-150 da, 149-250 da, etc.), and the last scan was for 999-2250 Da. During the MS scan, declustering potential (DP) and collision energy (CE) were maintained at -80V and -35±30V, respectively for ESI- and 50V and 35±30V. MS and MS/MS autocalibrations were performed using a calibrant delivery system (CDS) after every five injections to maintain mass accuracy (±5 ppm) and resolution (at least 10,000). Autocalibrations and full calibration after instrument maintenance used ESI- calibration solutions provided by the vendor (AB Sciex).

*QA/QC*

Method blanks, solvent (i.e., analytical) blanks, instrument sensitivity checks, and calibration verifications were used as quality control samples. IS recoveries were used to account for matrix effects and analyte losses during extraction. IS recoveries in unknown samples were calculated according to Equation S1 (**Eq S5**). Peaks of internal standards and calibrants (target analytes) in unknown samples were only considered for further analysis if retention times were ±30 s of calibration standards, signal to noise ratios were greater than 10 and also at least 3X higher than the response in instrument blanks. Each analytical run consisted of 14 calibration standards (0.5-5000 ng/L), method blanks, instrument blanks, instrument sensitivity checks (ISCs, 0.5-10 ng/L), low concentration continuing calibration verification (CCV, 10 ng/L), and mid-point CCV (200 ng/L). All quality control samples except instrument blanks contained 200 ng/L of each IS. Vial composition of all quality control samples was the same as unknown samples (30% methanol/70% water).

**Supplemental Equation S1.** Internal standard (IS) recovery

| $IS recovery \left( \% \right)=\left( \frac{IS peak area from unknown samples}{Average ISpeak area in calibration curve} \right)x100$ |  |
| --- | --- |

ISCs were performed by running 0.5-10 ng/L standards immediately prior to unknown samples. The limit of quantitation of an analyte was the lowest ISC where the calculated concentration was ±30% of true concentration or the concentration detected in the method blank, whichever was higher. CCV was performed by injecting a standard after every 10 unknown samples (alternating between 10 and 200 ng/L) and sample data were accepted only if CCVs were ±30% of true value. Calibration curves were fit with regression equations (R^2^>0.99) and used to quantify analytes in unknown samples. Every sample was quantified using an isotope dilution method, and concentrations of samples are reported as average of triplicates. Relative standard deviation (RSD) of replicates was calculated and presented as a measure of variability during the analysis.

**Supplemental Table S2.** Target analytes and their isotopically labeled internal standards (IS).

| **Per- and polyfluoroalkyl substance (PFAS) name** | **Acronym** | **IS** |
| --- | --- | --- |
| **Perfluoroalkanoic acids (PFCAs)** | | |
| Perfluorobutanoic acid | PFBA | [13C4] PFBA |
| Perfluoropentanoic acid | PFPeA | [13C5] PFPeA |
| Perfluorohexanoic acid | PFHxA | [13C5] PFHxA |
| Perfluoroheptanoic acid | PFHpA | [13C4] PFHpA |
| Perfluorooctanoic acid | PFOA | [13C8] PFOA |
| Perfluorononanoic acid | PFNA | [13C5] PFNA |
| Perfluorodecanoic acid | PFDA | [13C6] PFDA |
| Perfluoroundecanoic acid | PFUdA | [13C7]PFUnA |
| Perfluorododecanoic acid | PFDoA | [13C2] PFDoA |
| Perfluorotridecanoic acid | PFTrDA | [13C2] PFTeDA |
| Perfluorotetradecanoic acid | PFTeDA | [13C2] PFTeDA |
| **Perfluoroalkane Sulfonates (PFSAs)** | | |
| Perfluorobutane sulfonate | PFBS | [13C3] PFBS |
| Perfluoropentane sulfonate | PFPeS | [13C3] PFBS |
| Perfluorohexane sulfonate | PFHxS | [13C8] PFOS |
| Perfluoroheptane sulfonate | PFHpS | [13C8] PFOS |
| Perfluorooctane sulfonate | PFOS | [13C8] PFOS |
| Perfluorononane sulfonate | PFNS | [13C8] PFOS |
| Perfluorodecane sulfonate | PFDS | [13C8] PFOS |
| **Perfluoroalkane sulfonamides (FASAs)** | | |
| Perfluorobutane sulfonamide | FBSA | [13C8] FOSA |
| Perfluorohexane sulfonamide | FHxSA | [13C8] FOSA |
| Perfluorooctane sulfonamide | FOSA | [13C8] FOSA |
| Perfluorodecane sulfonamide | FDSA | [13C8] FOSA |
| N-methylperfluorooctane sulfonamide | N-MeFOSA-M | [2H3] N- MeFOSA-M |
| N-ethylperfluorooctane sulfonamide | N-EtFOSA-M | [2H3] N- EtFOSA-M |
| **Perfluoroalkane sulfonamido acetic acids** | | |
| N-methylperfluorooctane sulfonamido acetic acid | N-MeFOSAA | [2H3] N- MeFOSAA |
| N-ethylperfluorooctane sulfonamido acetic acid | N-EtFOSAA | [2H5] N-EtFOSAA |
| **Fluorotelomer Sulfonates (n:2 FTS)** | | |
| 4:2 fluorotelomer sulfonate | 4:2 FTS | [13C2] 4:2FTS |
| 6:2 fluorotelomer sulfonate | 6:2 FTS | [13C2] 6:2FTS |
| 8:2 fluorotelomer sulfonate | 8:2 FTS | [13C2] 8:2FTS |
| **Fluorotelomer Carboxylates (n:2/n:3 FTCAs)** | | |
| 3:3 fluorotelomer carboxilic acid | 3:3 FTCA | [13C2] 6:2FTCA |
| 5:3 fluorotelomer carboxilic acid | 5:3 FTCA | [13C2] 6:2FTCA |
| 7:3 fluorotelomer carboxilic acid | 7:3 FTCA | [13C2] 6:2FTCA |
| 6:2 fluorotelomer carboxilic acid | 6:2 FTCA | [13C2] 6:2FTCA |
| 8:2 fluorotelomer carboxilic acid | 8:2 FTCA | [13C2] 8:2FTCA |
| 10:2 fluorotelomer carboxilic acid | 10:2 FTCA | [13C2] 10:2FTCA |
| **Fluoroalkyl ether acids** | | |
| Sodium dodecafluoro-3H-4,8-dioxanonanoate | NaDONA | [13C8] PFOA |
| Hexafluoropropylene oxide dimer acid | HPFO-DA | [13C3]HFPO-DA |
| Perfluoro(2-ethoxyethane) sulfonic acid | PFEESA | [13C3] PFBS |
| Perfluoro-3-methoxypropanoic Acid | PFMPA | [13C3] PFBA |
| Perfluoro-4-methoxybutanoic Acid | PFMBA | [13C5] PFHxA |
| Perfluoro-3,6-dioxaheptanoic acid | NFDHA | [13C4] PFHpA |
| **Chloroperfluoroalkyl acids** | | |
| Sodium 8-chloroperfluoro-1-octansulfonate | 8Cl-PFOS | [13C8] PFOS |
| 9-Chlorohexadecafluoro-3-oxanonane--sulfonic acid | 9Cl-PF3ONS | [13C8] PFOS |
| 11-Chloroeicosafluoro-3-oxaundecane-1-sulfonic acid | 11Cl-PF3OUdS | [13C8] PFOS |
| **Zwitterionic PFAS** | | |
| N-(3-dimethylaminopropan-1-yl)perfluoro-1-hexanesulfonamide | N-AP-FHxSA | [2H3] N- MeFOSAA |
| 6:2 fluorotelomer sulfonamido propyl betaine | 6:2 FTSA PrB | [2H3] N- MeFOSAA |
| N-dimethyl ammonio propyl perfluorohexane sulfonamide | AmPr-FHxSA | [2H3] N- MeFOSAA |

**Supplemental Table S3.** Multiple reaction monitoring high resolution (MRM-HR) transitions and retention times of PFAS monitored in this study.

| **Analyte** | **Precursor (Q1)** | | **Quantifier (Q3)** | | **Qualifier (Q3)** | | **RT (min)** |
| --- | --- | --- | --- | --- | --- | --- | --- |
|  | **m/z (Da)** | **DP^2^**  **(V)** | **m/z**  **(Da)** | **CE^3^**  **(V)** | **m/z**  **(Da)** | **CE^3^ (V)** |  |
| PFBA^1^ | 212.98 | -25 | 168.9894 | -10 |  |  | 5.48 |
| [13C4] PFBA | 216.99 | -25 | 171.9994 | -10 |  |  | 5.48 |
| PFPeA^1^ | 262.98 | -25 | 218.9862 | -10 |  |  | 5.6 |
| [13C5] PFPeA | 267.99 | -25 | 222.9996 | -10 |  |  | 5.6 |
| PFBS | 298.94 | -55 | 79.9574 | -58 | 98.9558 | -40 | 5.62 |
| [13C3] PFBS | 301.95 | -55 | 79.9574 | -58 |  |  | 5.62 |
| PFHxA | 312.97 | -40 | 268.9830 | -10 | 118.9926 | -25 | 6.01 |
| [13C5] PFHxA | 317.99 | -40 | 272.9964 | -10 |  |  | 6.01 |
| 4:2 FTS | 326.97 | -35 | 306.9681 | -25 | 80.9652 | -65 | 5.97 |
| [13C2] 4:2FTS | 328.98 | -35 | 308.9748 | -65 | 79.9574 | -25 | 5.97 |
| PFPeS | 348.94 | -60 | 79.9574 | -66 | 98.9558 | -45 | 6.75 |
| PFHpA | 362.97 | -30 | 318.9798 | -15 | 168.9894 | -25 | 6.84 |
| [13C4] PFHpA | 366.98 | -30 | 321.9898 | -15 |  |  | 6.84 |
| PFOA | 412.97 | -60 | 368.9766 | -15 | 168.9894 | -25 | 7.46 |
| [13C8] PFOA | 420.99 | -60 | 376.0001 | -15 |  |  | 7.46 |
| 6:2 FTS | 427 | -25 | 406.9617 | -30 | 80.9652 | -55 | 7.44 |
| [13C2] 6:2FTS | 429 | -25 | 80.9652 | -55 |  |  | 7.44 |
| PFHpS | 449 | -65 | 79.9574 | -88 | 98.9558 | -50 | 7.45 |
| PFNA | 462.96 | -60 | 418.9734 | -15 | 168.9894 | -25 | 7.49 |
| [13C5] PFNA | 468 | -60 | 422.9868 | -15 |  |  | 7.49 |
| FOSA^1^ | 498 | -105 | 77.9655 | -70 |  |  | 8.5 |
| [13C8] FOSA | 506 | -105 | 77.9655 | -70 |  |  | 8.5 |
| [13C8] PFOS | 506.96 | -25 | 79.9574 | -108 |  |  | 7.97 |
| PFDA | 512.96 | -45 | 468.9702 | -15 | 168.9894 | -30 | 9.05 |
| [13C6] PFDA | 518.98 | -45 | 473.987 | -15 |  |  | 9.05 |
| 8:2 FTS | 527 | -25 | 506.9553 | -40 | 80.9652 | -55 | 8.05 |
| [13C2] 8:2FTS | 529 | -25 | 79.9574 | -55 |  |  | 8.05 |
| PFNS | 549 | -70 | 79.9574 | -110 | 98.9558 | -70 | 8.51 |
| PFUdA | 562.96 | -45 | 518.967 | -20 | 168.9894 | -30 | 9.58 |
| [13C7]PFUdA | 569.98 | -45 | 524.9871 | -20 |  |  | 9.58 |
| N-MeFOSAA | 570 | -25 | 418.9734 | -25 | 482.9353 | -25 | 10.05 |
| [2H3] N- MeFOSAA | 573 | -25 | 418.9734 | -25 |  |  | 10.05 |
| N-EtFOSAA | 584 | -75 | 418.9734 | -25 | 525.9775 | -30 | 8.62 |
| [2H5] N-EtFOSAA | 589 | -75 | 418.9734 | -25 |  |  | 8.62 |
| PFDS | 599 | -85 | 79.9674 | -118 | 98.9558 | -84 | 9.04 |
| PFDoA | 613 | -45 | 568.9638 | -20 | 168.9894 | -30 | 10.08 |
| [13C2] PFDoA | 615 | -45 | 569.9672 | -20 |  |  | 10.08 |
| PFTrDA | 663 | -30 | 618.9606 | -20 | 168.9894 | -35 | 10.53 |
| PFTeDA | 713 | -25 | 668.9574 | -25 | 168.9894 | -40 | 10.93 |
| [13C2] PFTeDA | 715 | -25 | 669.9608 | -25 |  |  | 10.93 |
| PFOS | 498.93 | -130 | 79.9574 | -65 | 98.9558 | -50 | 7.97 |
| PFHxS | 399 | -125 | 79.9574 | -55 | 98.9558 | -45 | 6.94 |
| FBSA | 298 | -55 | 77.9655 | -65 | 64 | -105 | 6.19 |
| FHxSA | 398 | -75 | 77.9655 | -150 | 64 | -150 | 7.3 |
| NaDONA | 377 | -30 | 85 | -35 | 251 | -20 | 6.51 |
| [13C3]HFPO-DA | 332 | -30 | 185 | -35 | 119 | -45 | 6.14 |
| HPFO-DA | 285 | -50 | 185 | -25 | 119 | -40 | 6.14 |
| 9Cl-PF3ONS | 531 | -30 | 351 | -45 | 83 | -95 | 8.45 |
| 11Cl-PF3OUdS | 631 | -55 | 451 | -50 | 83 | -145 | 9.22 |
| PFEESA | 315 | -80 | 135 | -40 | 69 | -60 | 5.8 |
| PFMPA | 229 | -25 | 85 | -45 | 135 | -15 | 5.28 |
| PFMBA | 279 | -35 | 85 | -10 | 185 | -20 | 5.71 |
| FDSA | 598 | -155 | 77.9655 | -55 | 578 | -40 | 9.64 |
| NFDHA | 295 | -25 | 85 | -25 | 201 | -20 | 5.94 |
| AmPr-FHxSA | 483 | -125 | 168.9894 | -40 | 319 | -30 | 7.67 |
| 6:2 FTSA PrB | 569 | -135 | 549 | -15 | 65 | -85 | 7.62 |
| 3:3 FTCA | 241 | -35 | 117 | -35 | 195 | -15 | 5.62 |
| 5:3 FTCA | 341 | -55 | 217 | -45 | 295 | -15 | 6.62 |
| 7:3 FTCA | 441 | -80 | 337 | -15 | 395 | -20 | 7.73 |
| 6:2 FTCA | 377 | -35 | 293 | -30 | 63 | -10 | 6.63 |
| 8:2 FTCA | 477 | -35 | 393 | -25 | 63 | -10 | 8.23 |
| 10:2 FTCA | 577 | -30 | 493 | -20 | 63 | -15 | 9.33 |
| [13C2] 6:2FTCA | 379 | -30 | 294 | -30 | 64 | -10 | 6.63 |
| [13C2] 8:2FTCA | 479 | -35 | 394 | -25 | 64 | -10 | 8.23 |
| [13C2] 10:2FTCA | 579 | -30 | 494 | -20 | 64 | -10 | 9.33 |
| 8Cl-PFOS | 515 | -25 | 98.9558 | -70 | 80 | -140 | 8.07 |
| N-MeFOSA-M | 512 | -140 | 168.9894 | -35 | 219 | -30 | 9.31 |
| [2H3] N- MeFOSA-M | 515 | -145 | 168.9894 | -40 | 219 | -35 | 9.31 |
| N-EtFOSA-M | 526 | -140 | 168.9894 | -35 | 219 | -35 | 9.66 |
| [2H3] N- EtFOSA-M | 531 | -145 | 219 | -35 | 168.9894 | -35 | 9.66 |

^1^Only one transition available for monitoring; ^2^Declustering potential (DP); ^3^Collision energy (CE).

**Supplemental Table S4.** Comparison of laboratory analytical list and limits of quantitation (LOQs)

|  |  |  |  |  |  |
| --- | --- | --- | --- | --- | --- |
| **CAS Number** | **Analyte** | **Texas Tech** | **Eurofins** | **Number of Carbon Atoms** | **Group** |
|  |  | *ng g^-1^ dw* | |  |  |
| 375-22-4 | PFBA | 1 | 0.05 | 4 | PFCA |
| 2706-90-3 | PFPeA | 0.5 | 0.1 | 5 |  |
| 307-24-4 | PFHxA | 0.2 | 0.05 | 6 |  |
| 375-85-9 | PFHpA | 0.1 | 0.05 | 7 |  |
| 335-67-1 | PFOA | 0.2 | 0.05 | 8 |  |
| 375-95-1 | PFNA | 1 | 0.05 | 9 |  |
| 335-76-2 | PFDA | 0.2 | 0.05 | 10 |  |
| 2058-94-8 | PFUnA | 0.2 | 0.05 | 11 |  |
| 307-55-1 | PFDoA | 0.2 | 0.05 | 12 |  |
| 72629-94-8 | PFTrDA | 0.5 | 0.05 | 13 |  |
| 376-06-7 | PFTeDA | 1 | 0.05 | 14 |  |
| 375-73-5 | PFBS | 0.2 | 0.05 | 4 | PFSA |
| 2706-91-4 | PFPeS | 2 | 0.05 | 5 |  |
| 335-46-4 | PFHxS | 2 | 0.05 | 6 |  |
| 375-92-8 | PFHpS | 2 | 0.05 | 7 |  |
| 1763-23-1 | PFOS | 0.2 | 0.05 | 8 |  |
| 1651215-26-7 | 8Cl-PFOS | 2 |  | 8 |  |
| 474511-07-4 | PFNS | 2 | 0.05 | 9 |  |
| 335-77-3 | PFDS | 1 | 0.05 | 10 |  |
| 79780-39-5 | PFDoS | - | 0.05 | 12 |  |
| 377-73-1 | PFMPA | 2 | 0.1 | 4 | PFECA |
| 863090-89-5 | PFMBA | 2 | 0.1 | 5 |  |
| 151772-58-6 | NFDHA | 2 | 0.1 | 5 |  |
| 13252-13-6 | HFPO-DA | 0.5 | 0.05 | 6 |  |
| 919005-14-4 | ADONA | 1 | 0.2 | 7 |  |
| 113507-82-7 | PFEESA | 1 | 0.1 | 4 | PFESA |
| 756426-58-1 | 9Cl-PF3ONS | 0.1 | 0.2 | 8 |  |
| 763051-92-9 | 11Cl-PF3OUdS | 2 | 0.2 | 10 |  |
| 30334-69-1 | PFBSA | 2 |  | 4 |  |
| 41997-13-1 | PFHxSA | 2 |  | 6 |  |
| 754-91-6 | PFOSA | 0.2 | 0.05 | 8 |  |
| 31506-32-8 | N-MeFOSA | 2 | 0.05 | 9 |  |
| 4151-50-2 | N-EtFOSA | 2 | 0.05 | 10 |  |
| 4262-70-8 | PFDSA | 1 |  | 10 |  |
| 50598-28-2 | N-Ap-FHxSA | 2 |  | 11 |  |
| 70248-51-0 | AmPr-FHxSA | 2 |  | 12 |  |
| 2355-31-9 | N-MeFOSAA | 0.5 | 0.05 | 9 | FASAA |
| 2991-50-6 | N-EtFOSAA | 0.5 | 0.05 | 12 |  |
| 24448-09-7 | N-MeFOSE | - | 0.5 | 11 | FASE |
| 1691-99-2 | N-EtFOSE | - | 0.5 | 12 |  |
| 356-02-5 | 3:3 FTCA | 2 | 0.25 | 6 | FTCA |
| 914637-49-3 | 5:3 FTCA | 2 | 1 | 8 |  |
| 53826-12-3 | 6:2 FTCA | 2 |  | 8 |  |
| 812-70-4 | 7:3 FTCA | 2 | 1 | 10 |  |
| 27854-31-5 | 8:2 FTCA | 2 |  | 10 |  |
| 53826-13-4 | 10:2 FTCA | 2 |  | 12 |  |
| 757124-72-4 | 4:2 FTS | 1 | 0.2 | 6 | FTS |
| 27619-97-2 | 6:2 FTS | 0.5 | 0.35 | 8 |  |
| 39108-34-4 | 8:2 FTS | 0.5 | 0.35 | 10 |  |
| 34455-29-3 | 6:2 FTSA PrB | 2 |  | 15 | FTSAB |

*Soil analysis methods (non-PFAS)*

Soil texture and mineral analyses used at the University of Minnesota Research Analytical Lab (UMRAL). Full soil analysis datasets are presented in **Table S2**.

Soil texture

Miller, R.O., R. Gavlak, D. Horneck. 2013. Particle Size Analysis (Hydrometer). p. 95-98. Soil, Plant, and Water Reference Methods for the Western Region. 4th Ed.

Total organic carbon

*Method 5310 Total Organic Carbon (TOC). p.5:10-15 of:*

American Public Health Association, American Water Works Association, Water Environment Federation. 2017. Standard methods for the examination of water and wastewater. 34rd ed. Am. Public Health Assoc., 800 I Street, Washington, DC 20001.

Organic matter, soil pH, P, S, B, Zn, Cu, Fe, and Mn

Recommended Chemical Soil Test Procedures for the North Central Region. North Central Regional Research Publication No. 221 (Revised). Jan. 1998. Missouri Agricultural Experiment Station SB 1001.

K, Ca, Mg, Na

Thomas, G. W. Exchange Cations. Method 9-3.1. p.159-165. In the reference below:

Page, A. L. (ed.) 1982. Methods of soil analysis. Part 2. Chemical and Microbiological Properties, 2nd Ed., ASA, SSA, Madison, WI 53711.

Fassel, V.A., and R.N. Kniseley. Nov. 1974. Inductively Coupled Plasma Optical Emission Spectroscopy. Anal. Chem. 46 (13):1110A-1120A. Also: Dahlquist, R.L. and J.W. Knoll. 1978. Inductively Coupled Plasma-Atomic Emission Spectrometry: Analysis of biological materials and soils for major trace, and ultra-trace elements. Appl. Spectroscopy 32:1-30. ICP: ARL (Fisons) Model 3560 ICP-AES. **Using the equipment below:**

Thermo Instrument Systems Inc. (Fisons Instruments Inc. Division) 81 Wyman Street PO Box 9046 Waltham, MA 02254.

**Supplemental Table S5.** The datasets generated during and/or analyzed during the current study are available in the MPCA File Server repository: <https://files.pca.state.mn.us/pub/file_requests/datasets/PFAS/Table_S5_PFAS_in_Land-Applied_Biosolids_Streets.xlsx>

*Human health risk assessment*

The purpose of this risk assessment is to estimate human health exposure risks from consumption of animals that were exposed to perfluorooctane sulfonate (PFOS) due to land-application of biosolids. The assessment considers exposure risks by estimating PFOS uptake into cattle that are exposed to feed grown on land where biosolids were applied and subsequent consumption of the cattle (beef and milk) by children and adults (Figure S6). The assessment relies on Site I soil data (field 1) and corn stover data (field 3). This assessment is focused on PFOS only because it is one of the most bioaccumulative PFAS and is typically detected at the highest concentrations in biosolids when compared to other PFAS. The assessment considers exposure risks by estimating PFOS uptake into cattle that are exposed to feed grown on land where biosolids were applied and subsequent consumption of the cattle (beef and milk) by children and adults.

Perfluorooctanoic acid (PFOA) is also frequently detected in biosolids but does not tend to bioaccumulate in cattle (Vestergren et al., 2013, Mikkonen et al., 2023a). Given PFOA’s toxicity (MDH 2024a), even relatively small amounts of PFOA in the environment can theoretically lead to unacceptable risk estimates. However, with feed being the primary source of PFAS exposure to cattle under this risk scenario, PFOA was excluded from the assessment because it was not detected in plant samples (corn stover) that factor into this assessment.


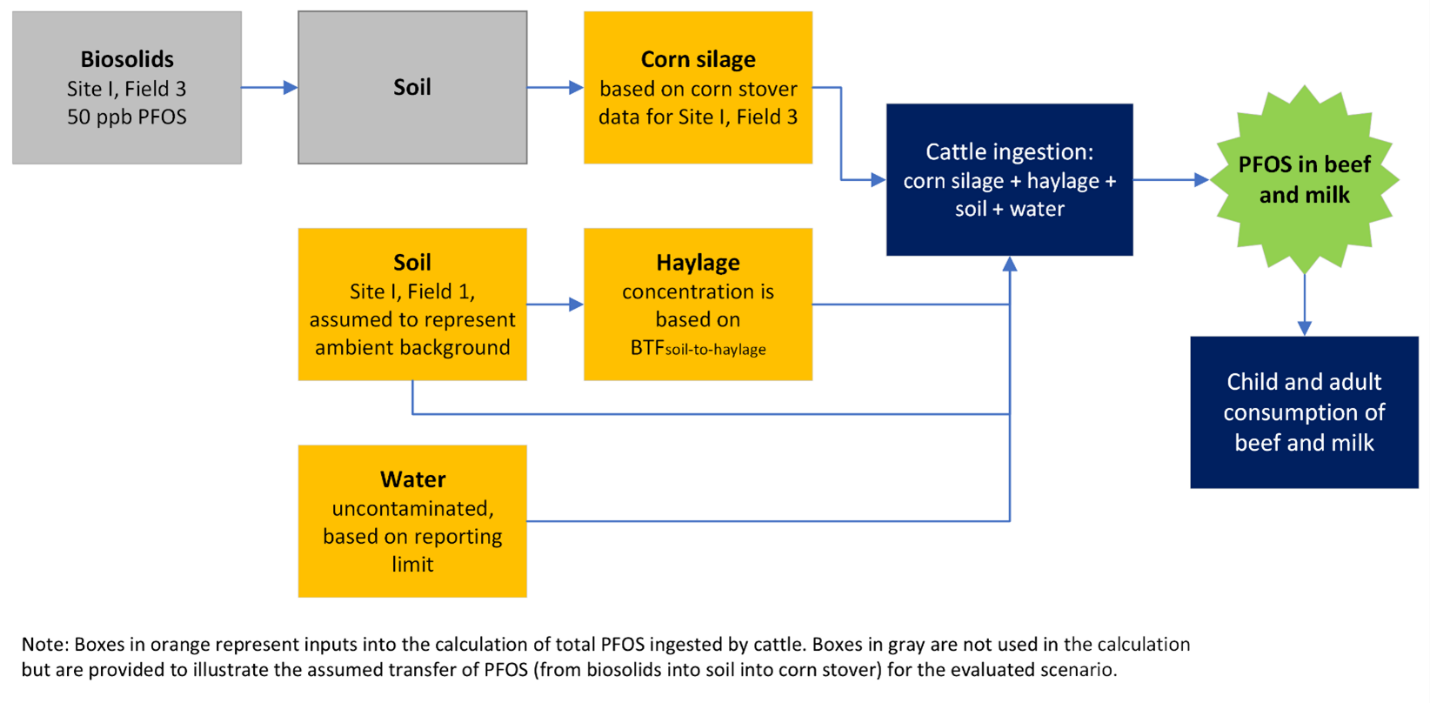


**Supplemental Figure S2.** Simple exposure model for estimating PFOS concentration in beef muscle and milk. The PFOS soil concentration used in the model was the mean PFOS soil concentration at location I, field 3. The water was assumed to be uncontaminated above the method reporting limit.

Haylage PFOS concentration was estimated based on PFOS transfer from soil to haylage using a soil-to-plant BTF (Equations S2 – S4). While corn silage is typically composed of stover and ears, corn ear samples for location I, field 3 were all non-detect for PFOS and were therefore excluded from the assessment. An estimate of the average PFOS stover + ears concentration for use in the risk calculations would not have been reliable given the very small sample size (n = 6) with 50% non-detects. Therefore, this assessment assumes that the silage is composed entirely of corn stover. The total exposure to the animal also included ingestion of water and incidental ingestion of soil while grazing. For this scenario, cattle were assumed to graze on a field with no history of biosolids application – location I, field 1 soil data were used in this assessment. Given that this field had no prior history of biosolids application, the soil concentrations were assumed to be representative of ambient background levels. Similarly, water was assumed to be uncontaminated and not impacted by biosolids application. The concentration used represents the lowest reporting limit (1.5 ng/L) for PFOS in water (Table S6; MPCA 2024).

The risk to humans was then estimated based on children (ages 1 to <6) and adults (age 20+) consuming beef and milk using mean and 90^th^ percentile consumption rates for both groups (Table S6). Both cancer and noncancer toxicity values are available (Minnesota Department of Health (MDH) 2024), which can be used to calculate PFOS beef and milk screening levels. The beef or milk screening level represents the maximum amount of PFOS in beef or milk that people can safely consume. Screening levels based on a target cancer risk of 1 in 100,000 (1 x 10^-5^, the default acceptable risk level in Minnesota) are less stringent than screening levels based on noncancer endpoints. Noncancer screening levels are therefore more health protective. As such, only noncancer risk estimates (hazard quotients) are provided and discussed

**Supplemental Table S6.** Model parameters and inputs

| **Exposure Parameter or model input** | **Value** | **Notes/Reference** |
| --- | --- | --- |
| Receptors | Child 1 to <6 years  Adult 20+ years |  |
| Consumption rates | g/kg BW-day | [Maine CDC 2020](https://www.maine.gov/dep/spills/topics/pfas/PFOS-Action-Levels-for-Beef-Derivation-Memo-08.04.20.pdf) and [Maine CDC 2017](https://www.maine.gov/dep/spills/topics/pfas/Derivation-of-Action-Levels-for-PFOS-in-Cows-Milk-03.28.17.pdf) |
| Child (1 to <6) | Mean = 2.3 (beef), 30.9 (milk)  90^th^ percentile = 4.7 (beef), 58.9 (milk) | The beef consumption rates for women of childbearing age (13 to 49) were slightly lower than for the adult (20+) population, therefore, screening levels developed for the adult population will also be protective of women of childbearing age ([Maine CDC 2020](https://www.maine.gov/dep/spills/topics/pfas/PFOS-Action-Levels-for-Beef-Derivation-Memo-08.04.20.pdf)). Milk consumption rates for the child receptor are an average of the 1-2 and 3-5 age groups as provided in [Maine CDC 2017](https://www.maine.gov/dep/spills/topics/pfas/Derivation-of-Action-Levels-for-PFOS-in-Cows-Milk-03.28.17.pdf). |
| Adult (20+) | Mean = 1.1 (beef), 2.7 (milk)  90^th^ percentile = 2.2 (beef), 6.1 (milk) |  |
| PFOS reference dose (RfD) | 1.0 ng/kg-d | [MDH 2024b](https://www.health.state.mn.us/communities/environment/risk/docs/guidance/gw/pfos.pdf) |
| Relative source contribution (RSC) | 0.8 | [Maine CDC 2020](https://www.maine.gov/dep/spills/topics/pfas/PFOS-Action-Levels-for-Beef-Derivation-Memo-08.04.20.pdf) |
| PFOS cancer slope factor (CSF)^1^ | 13 per mg/kg-d | [MDH 2024b](https://www.health.state.mn.us/communities/environment/risk/docs/guidance/gw/pfos.pdf) |
| **Cattle exposure assumptions** | **Value** | **Notes/Reference** |
| Feed ingested (beef cattle) | 29 kg WW/day split as:  14.5 kg/day of corn silage  14.5 kg/day of haylage | [USEPA PRG](https://epa-prgs.ornl.gov/radionuclides/users_guide.html) default is 11.77 kg DW/day; converted to WW assuming 60% moisture content. Feed composition varies according to cattle age; 50/50 split is assumed in this assessment. |
| Feed ingested (dairy cattle) | 51 kg WW/day split as:  25.5 kg/day of corn silage  25.5 kg/day of haylage | [USEPA PRG](https://epa-prgs.ornl.gov/radionuclides/users_guide.html) default is 20.3 kg DW/day; converted to WW assuming 60% moisture content. Feed composition varies according to cattle age; 50/50 split is assumed in this assessment. |
| Water ingested (beef cattle) | 53 L/day (or kg/day) | [USEPA PRG](https://epa-prgs.ornl.gov/radionuclides/users_guide.html) default |
| Water ingested (dairy cattle) | 92 L/day (or kg/day) | [USEPA PRG](https://epa-prgs.ornl.gov/radionuclides/users_guide.html) default |
| Soil ingested (beef cattle) | 0.5 kg/day | [USEPA PRG](https://epa-prgs.ornl.gov/radionuclides/users_guide.html) default; assumes ingestion of soil while grazing |
| Soil ingested (dairy cattle) | 0.4 kg/day | [USEPA PRG](https://epa-prgs.ornl.gov/radionuclides/users_guide.html) default; assumes ingestion of soil while grazing |
| Fraction of year  for cattle grazing | 0.5 | Cattle could be grazing May through October – 50% of the year ([UMN 2018](https://extension.umn.edu/forage-harvest-and-storage/plan-your-forage-supply-summer-grazing)). |
| **Biotransfer factors** | **Value** | **Notes/Reference** |
| PFOS BTF_soil-to-plant_ | Haylage = 0.02 WW veg/DW soil | Average of TFs reported in [Yoo et al. 2011](https://doi.org/10.1021/es102972m), [Mikkonen et al. 2023b](https://doi.org/10.1016/j.envint.2023.108218), and [Chou et al. 2023](https://doi.org/10.1016/j.fct.2023.114062). Converted to WW assuming 60% moisture content ([MSU 2011](https://www.canr.msu.edu/news/understanding_how_hay_dries_in_the_field#:~:text=Warm%20air%20temperature%20and%20low,move%20up%20into%20the%20swath.)). |
| PFOS BTF_beef muscle_ | 0.07 day/kg | [Vestergren et al. 2013](https://link.springer.com/article/10.1007/s11356-013-1722-x) |
| PFOS BTF_milk_ | 0.02 day/kg | [Vestergren et al. 2013](https://link.springer.com/article/10.1007/s11356-013-1722-x) |
| **Env. media concentrations** | **Value** | **Notes/Reference** |
| Water | 1.5 ng/L | Based on lowest reporting limit ([MPCA 2024](https://www.pca.state.mn.us/sites/default/files/tdr-g1-25.pdf)), assumes water is uncontaminated. |
| Soil | 0.12 ng/g DW | Considered to be an ambient background concentration (no history of biosolids application), based on highest detected concentration for Site I, Field 1. |
| Corn stover | 0.15 ng/g WW | Corn stover average concentration (n = 3) for Site I, Field 3 is 0.21 ng/g dw, converted to WW assuming 30% moisture content ([MSU 2007](https://www.canr.msu.edu/uploads/236/58572/CornStoverHarvesting.pdf)). |

**1 – The PFOS CSF was used to calculate screening levels. However, the levels based on the cancer endpoint were less stringent than for noncancer. Therefore, only noncancer risk estimates are provided.**

*Biotransfer factors*

BTFs are used to estimate contaminant concentrations in vegetation and animals if a direct measurement of the plant or animal tissue is not available. They are derived using simple empirical equations which relate an environmental media concentration to a biota concentration. For plants, the BTF represents the ratio of the contaminant concentration in fresh weight plant material (in mg/kg, for example) to the contaminant concentration in dry weight soil (in mg/kg, for example). For beef or milk, the BTF represents the ratio of the contaminant concentration in fresh weight tissue/meat/milk (in mg/kg, for example) to the daily intake of the contaminant by the animal (in mg/day, for example). For PFOS the BTFs are calculated as follows:

**Supplemental Equation S2. Soil-to-plant BTF**

$\boldsymbol{BTF}_{\boldsymbol{soil-to-plant}}=\frac{PFOS concentration in plant (wet weight)}{PFOS concentration in soil (dry weight)}$

**Equation S3. BTF to beef muscle.**

$$\boldsymbol{BTF}_{\boldsymbol{beef}}=\frac{PFOS concentration in beef muscle (wet weight)}{Daily intake of PFOS}$$

**Equation S4. BTF to cow milk.**

$\boldsymbol{BTF}_{\boldsymbol{milk}}=\frac{PFOS concentration in milk}{Daily intake of PFOS}$

*Noncancer assessment*

A simple schematic of the calculations is provided in Figure S8. The beef or milk screening level represents the maximum amount of PFOS in beef or milk that people can safely eat or drink. This approach does not account for any other human exposure pathways, such as through the consumption of other animal products that may be contaminated, or exposure to other PFAS. However, the calculation of the screening levels does include a relative source contribution (RSC) factor, which represents the fraction of exposure coming from a particular pathway and is an indirect way of accounting for other sources of contaminant exposure. In this way RSCs provide a margin of safety to account for the possibility of exposure through other sources.


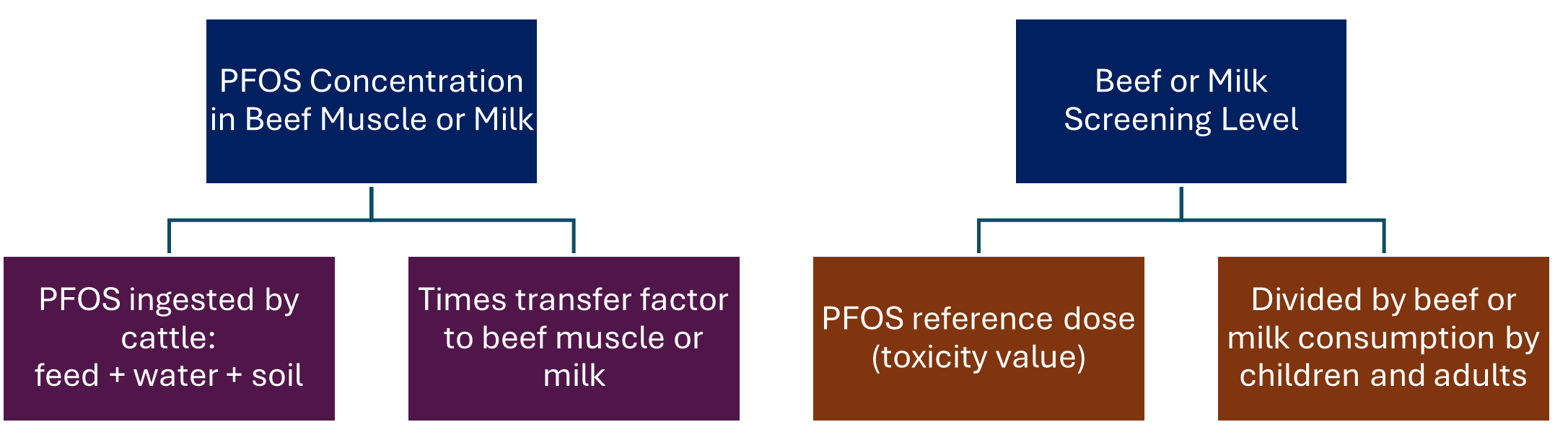


**Supplemental Figure S3.** Simple schematic showing the calculation of PFOS concentration in beef muscle or milk, and PFOS screening levels.

Equations for calculation of the following are also provided:

- Concentration of PFOS in beef muscle and milk based on cattle ingestion of feed, water, and soil
- PFOS screening levels for beef and milk
- PFOS hazard quotients based on human consumption of beef and milk

**Supplemental Equation S3a and S3b. Concentration of PFOS in beef muscle (a) and milk (b)**

$\boldsymbol{C}_{\boldsymbol{beef muscle}}=\left[ \left( {IR}_{corn silage}\times C_{corn stover} \right)+\left( {IR}_{haylage}\times{BTF}_{soil-to-haylage}\times C_{soil} \right)+\left( {IR}_{water}\times C_{water} \right)+\left( {IR}_{soil}\times C_{soil}\times F_{grazing} \right) \right]\times{BTF}_{beef}$

$\boldsymbol{C}_{\boldsymbol{milk}}=\left[ \left( {IR}_{corn silage}\times C_{corn stover} \right)+\left( {IR}_{haylage}\times{BTF}_{soil-to-haylage}\times C_{soil} \right)+\left( {IR}_{water}\times C_{water} \right)+\left( {IR}_{soil}\times C_{soil}\times F_{grazing} \right) \right]\times{BTF}_{milk}$

Where:

C_beef muscle_ = PFOS concentration in beef muscle in ng/kg WW

C_milk_ = PFOS concentration in milk in ng/kg WW

IR_corn silage_ = cattle intake rate of corn silage in kg WW/day

C_corn stover_ = PFOS concentration in corn stover in ng/kg WW

IR_haylage_ = cattle intake rate of haylage in kg WW/day

BTF_soil-to-haylage_ = PFOS BTF for soil to haylage in WW veg/DW soil

C_soil_ = PFOS concentration in soil in ng/kg DW

IR_water_ = cattle intake rate of water in L/day or kg/day

C_water_ = PFOS concentration in water in ng/L or ng/kg

IR_soil_ = cattle intake rate of soil in kg DW/day

F_grazing_ = fraction of time that cattle are grazing (unitless)

BTF_beef_ = BTF for PFOS to beef muscle in day/kg

BTF_milk_ = BTF for PFOS to milk in day/kg

**Supplemental Equation S4. Screening level for beef or milk**

$\boldsymbol{SL}_{\boldsymbol{beef or milk}}=\frac{PFOS RfD}{{CR}_{child or adult}}\times RSC\times CF$

Where:

SL_beef or milk_ = screening level in beef or milk in ng/kg

RfD = reference dose

CR_child or adult_ = child or adult consumption rate of beef or milk in g/kg BW-day

RSC = relative source contribution factor (unitless)

CF = conversion factor (1000 g/kg)

**Supplemental Equation S5. Hazard quotient**

$$\boldsymbol{HQ}=\frac{C_{beef or milk}}{{SL}_{beef or milk}}$$

Where:

HQ = hazard quotient (unitless)

C_beef or milk_ = PFOS concentration in beef muscle or milk in ng/kg WW

SL_beef or milk_ = PFOS screening level for beef or milk in ng/kg WW

Ultimately, steps to reduce sources of PFAS to wastewater and biosolids must be taken to reduce risk to consumers. Source reduction can be achieved through prohibitions on PFAS manufacturing and/or use similar to the recent prohibitions enacted in Minnesota (Minnesota Statute Section 116.943); regular testing of biosolids coupled with risk-based standards that set limits for land application; and increased testing of crops and animal products (i.e. meat and milk) produced on farms that have received PFAS-contaminated biosolids. Field studies evaluating management strategies for PFAS-impacted biosolids that consider soil type, depth to groundwater, and distance to surface waters will also be valuable in shaping best management practice. Choosing not to land apply biosolids in favor of other conventional management options such as incineration or landfilling comes with its own risks, including contamination of air and groundwater. Source reduction is paramount, as PFAS treatment and destruction in wastewater and biosolids can be prohibitively expensive (Ling et al., 2024).

When developing biosolids management plans, policy makers and local wastewater authorities should consider evaluating biosolid treatment technologies with the potential to destroy PFAS (Xue et al., 2025). Emerging biosolids management strategies cannot only destroy PFAS and other organic molecules of concern, but they have the potential to be more cost-effective than current biosolids management strategies (Keller et al., 2024).

*Additional figures and tables*


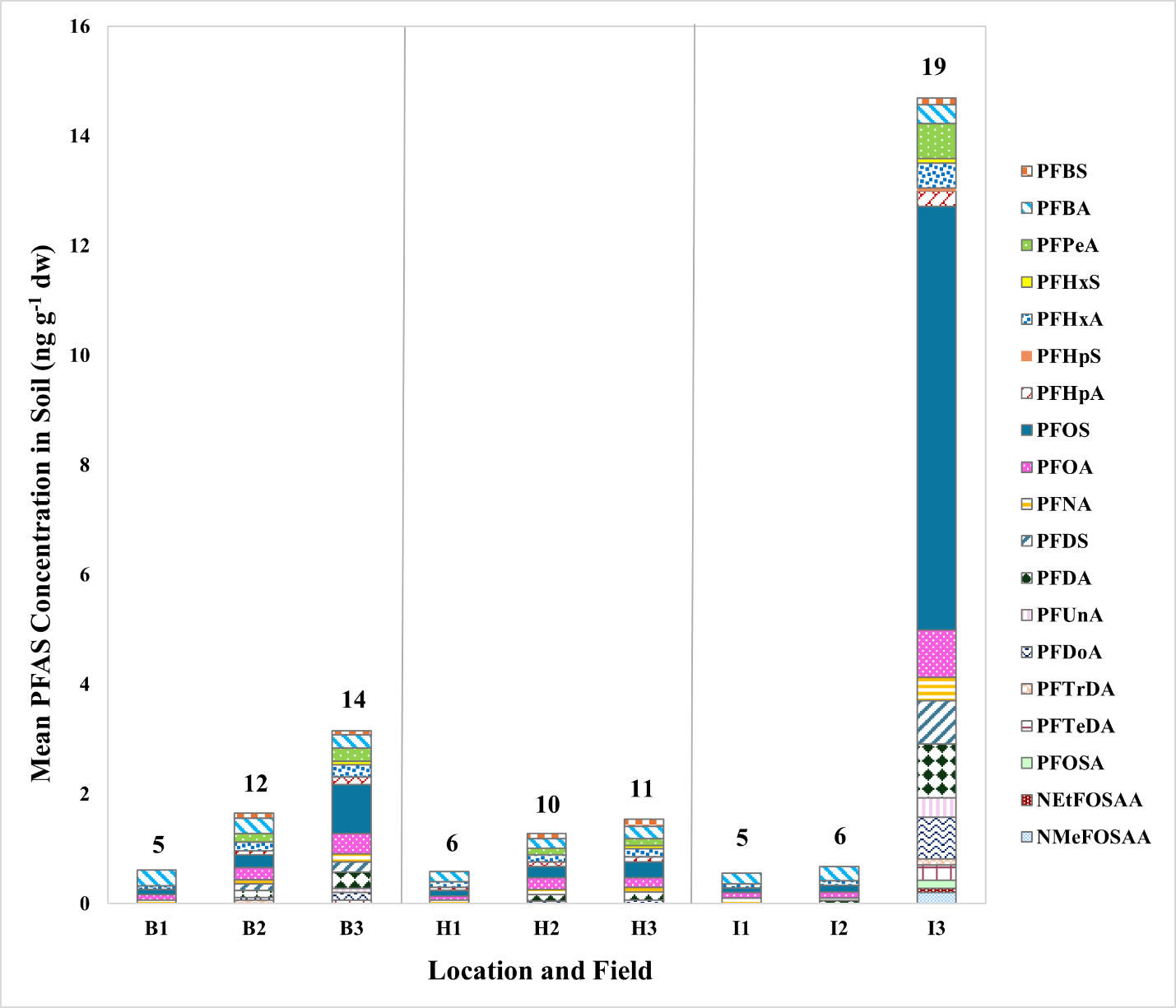


**Supplemental Figure S4.** Mean ƩPFAS concentration and number of PFAS detected in soil samples at each location and field (all dates and depths, combined). The number above each column is the number of PFAS detected at that field. Field 1 at all locations had no history of biosolids application. Field 2 at all locations had biosolids applied at least 2 years prior to this study. Field 3 received biosolids during the study year. Compared to the mean concentration of the nine focal PFAS (Fig. 1), the mean concentration of ƩPFAS at each location and field was significantly different (p < 0.05) at sites B3 and I3, but there was no significant difference in mean concentration at all other fields and locations demonstrating that the nine focal PFAS drive ƩPFAS.


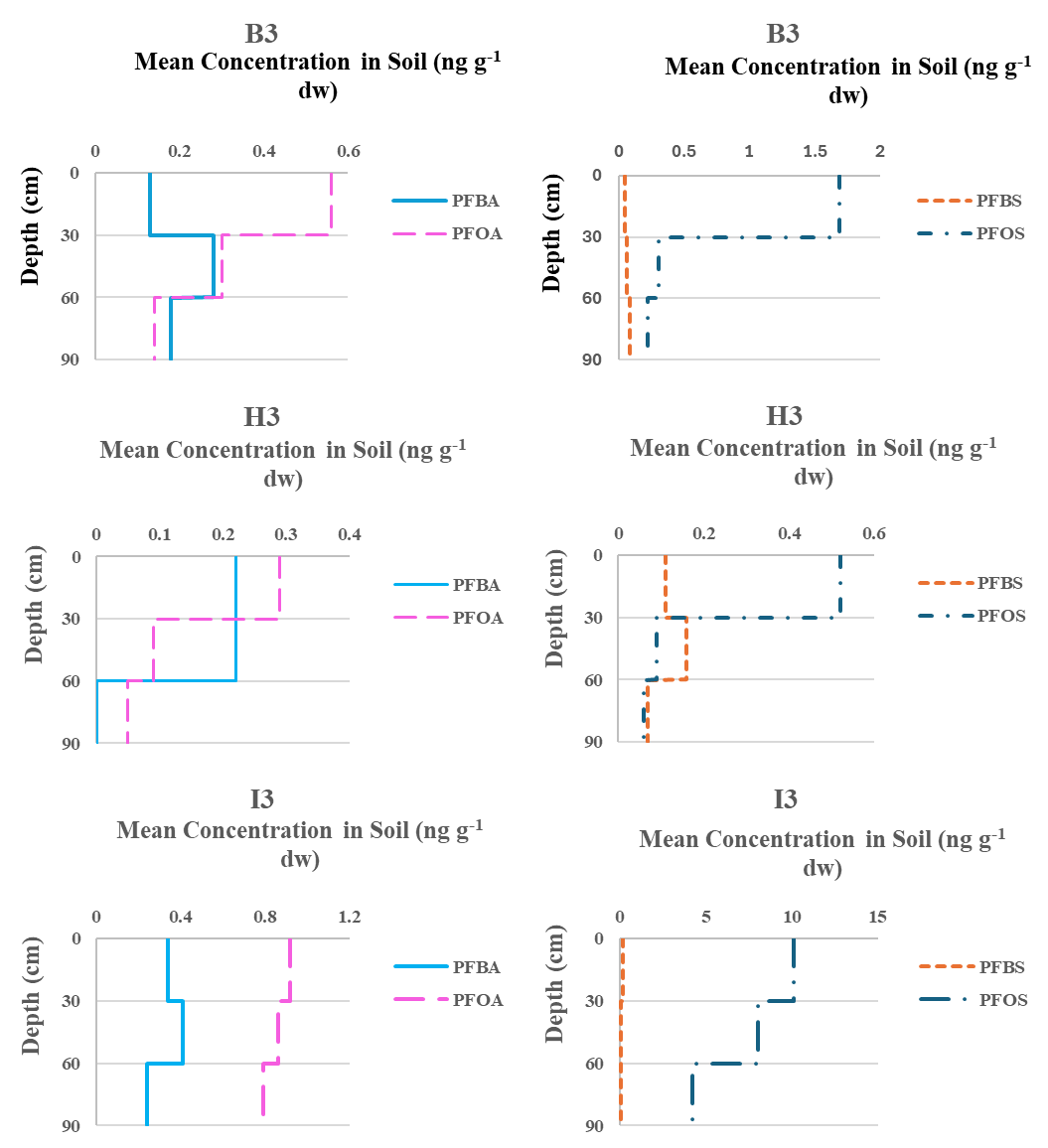


**Supplemental Figure S5**. Change in concentration of PFAS with depth in the soil profile in field 3 at each location. Soil mobility of a four-carbon carboxylate (PFBA) is greater than that of PFOA, an eight-carbon carboxylate. Likewise, mobility of a four-carbon sulfonate (PFBS) is greater than that of PFOS, an eight-carbon sulfonate, demonstrating the impact of chain length on soil mobility.


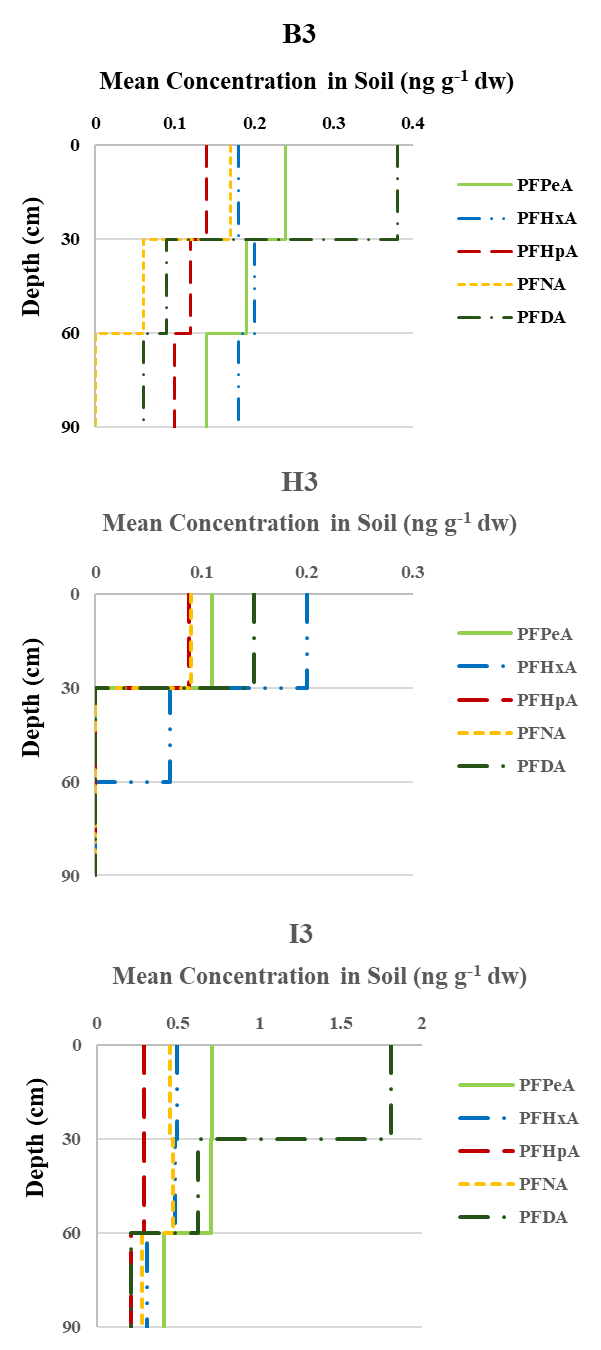


**Supplemental Figure S6**. Change in concentration with depth in the soil profile for C5 – C10 carboxylates in field 3 at each location. Longer chain carboxylates (e.g. PFDA) appear to be more strongly retained in the surficial soil layer compared to shorter-chain carboxylates.


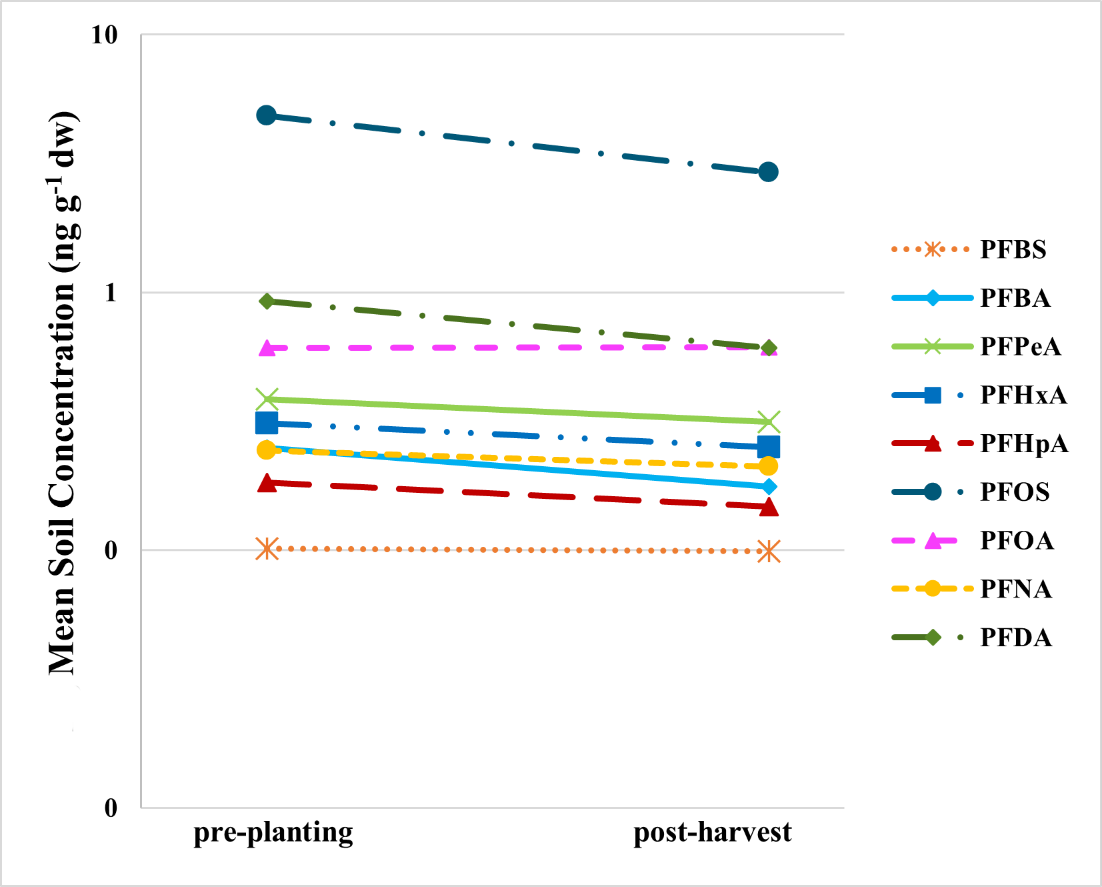


**Supplemental Figure S7**. Change in mean concentration of nine focal PFAS (all field 3 locations combined) in surficial soil (0-30 cm) over the course of a single growing season. The concentration of most PFAS changed slightly, or not at all, over the course of the growing season, and changes were not statistically significant (p > 0.05).


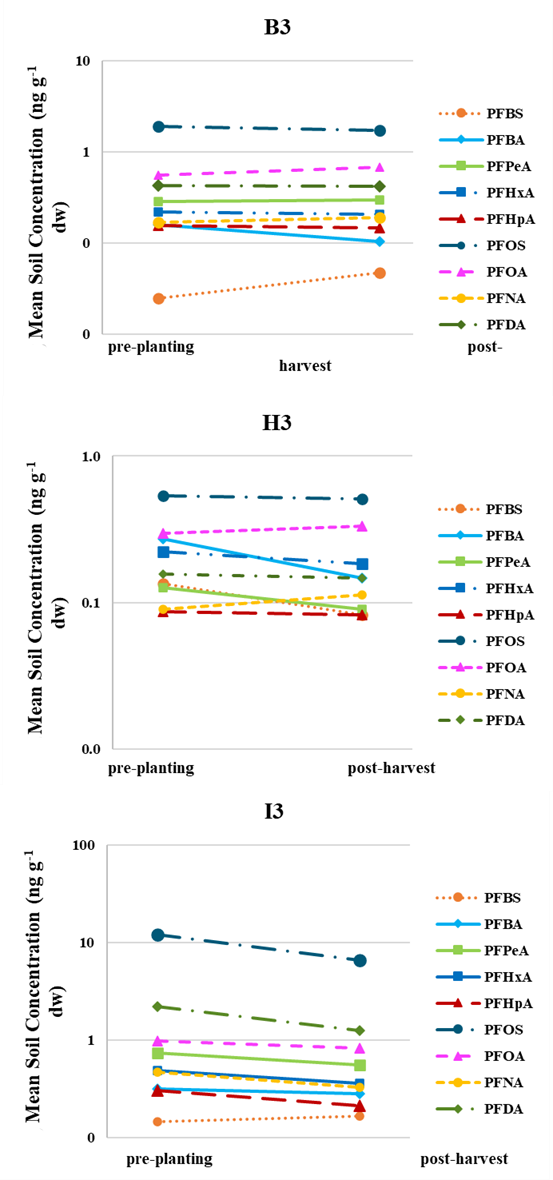


**Supplemental Figure S8**. Change in mean concentration of nine focal PFAS in field 3 at each location in surficial soil (0-30 cm) over the course of a single growing season. The concentration of most PFAS did not change, or changed only slightly, over the course of the growing season, and changes were not statistically significant (p > .05).

**Supplemental Table S7.** PFAS concentrations in corresponding crop and soil samples and bioaccumulation factors (BAFs).

|  |  |  | *Analyte* | | | | |
| --- | --- | --- | --- | --- | --- | --- | --- |
|  |  |  | **PFMPA** | **PFBS** | **PFBA** | **PFPeA** | **PFOS** |
|  |  |  | *ng g^-1^ dw (sd)* | | | | |
| Location & field | **B1** | Rye | nd | nd | 0.16 (0.01) | nd | nd |
|  |  | Soil | nd | nd | 0.28 (0.10) | nd | 0.08 (0.02) |
|  |  | BAF (unitless) | NA | NA | 0.57 | NA | NA |
|  |  |  |  |  |  |  |  |
|  | **B3** | Corn Stover | 1.0 (0.35) | 0.31 (0.075) | nd | nd | nd |
|  |  | Soil | nd | 0.06 (0.01) | 0.18 (0.08) | 0.20 (0.06) | 0.79 (0.34) |
|  |  | BAF (unitless) | NA | 5.2 | NA | NA | NA |
|  |  |  |  |  |  |  |  |
|  | **H1** | Corn Stover | 0.13 (0.07) | nd | nd | nd | nd |
|  |  | Soil | nd | nd | 0.19 (0.18) | nd | 0.09 (0.02) |
|  |  | BAF (unitless) | NA | NA | NA | NA | NA |
|  |  |  |  |  |  |  |  |
|  | **H3** | Corn Stover | 0.62 (0.50) | nd | 1.7 (1.4) | nd | nd |
|  |  | Soil | nd | 0.12 (0.06) | 0.22 (0.05) | nd | 0.30 (0.23) |
|  |  | BAF (unitless) | NA | NA | 7.7 | NA | NA |
|  |  |  |  |  |  |  |  |
|  | **I3** | Corn Stover | 0.30 (0.19) | 0.60 (0.06) | 2.4 (1.9) | 0.21 (0.10) | 0.21 (0.15) |
|  |  | Soil | nd | 0.12 (0.05) | 0.34 (0.09) | 0.62 (0.18) | 7.7 (3.2) |
|  |  | BAF (unitless) | NA | 5.0 | 7.0 | 0.34 | 0.03 |

*Supplemental References*

AWWA. (2018a). Standard Methods Committee of the American Public Health Association, American Water Works Association, and Water Environment Federation. (2018b). 4500-n nitrogen. In W. C. Lipps, T. E. Baxter, & E. Braun-Howland (Eds.), *Standard Methods For the Examination of Water and Wastewater*. APHA Press. https://doi.org/10.2105/SMWW.2882.086

AWWA. (2018b). Standard Methods Committee of the American Public Health Association, American Water Works Association, and Water Environment Federation. 2540 solids. In W. . Lipps, T. E. Baxter, & E. Braun-Howland (Eds.), *Standard Methods For the Examination of Water and Wastewater*. APHA Press. <https://doi.org/10.2105/SMWW.2882.030>Brown, J. R. (1998). *Recommended chemical soil test procedures for the North Central Region* (No. 1001). Missouri Agricultural Experiment Station, University of Missouri--Columbia.

Chou, W. C., Tell, L. A., Baynes, R. E., Davis, J. L., Cheng, Y. H., Maunsell, F. P., ... & Lin, Z. (2023). Development and application of an interactive generic physiologically based pharmacokinetic (igPBPK) model for adult beef cattle and lactating dairy cows to estimate tissue distribution and edible tissue and milk withdrawal intervals for per-and polyfluoroalkyl substances (PFAS). *Food and Chemical Toxicology*, *181*, 114062.

Fassel, V. A., & Kniseley, R. N. (1974). Inductively coupled plasma. Optical emission spectroscopy. *Analytical Chemistry*, *46*(13), 1110A-1120a

Guelfo, J.L., Higgins, C.P., 2013. Subsurface Transport Potential of Perfluoroalkyl Acids at Aqueous Film-Forming Foam (AFFF)-Impacted Sites. Environ. Sci. Technol. 47, 4164–4171. <https://doi.org/10.1021/es3048043>

Keller, A. A., Li, W., Floyd, Y., Bae, J., Clemens, K. M., Thomas, E., ... & Adeleye, A. S. (2024). Elimination of microplastics, PFAS, and PPCPs from biosolids via pyrolysis to produce biochar: Feasibility and techno-economic analysis. *Science of The Total Environment*, *947*, 174773.

Ling, A. L., Vermace, R. R., McCabe, A. J., Wolohan, K. M., & Kyser, S. J. (2024). Is removal and destruction of perfluoroalkyl and polyfluoroalkyl substances from wastewater effluent affordable?. *Water environment research*, *96*(1), e10975.

Maine CDC 2017. Action levels for PFOS in cow’s milk. Maine Center for Disease Control and Prevention. March 2017. <https://www.maine.gov/dep/spills/topics/pfas/Derivation-of-Action-Levels-for-PFOS-in-Cows-Milk-03.28.17.pdf>

Maine CDC 2020. Action levels for PFOS in beef for use in determining whether beef at a farm is adulterated. Maine Center for Disease Control and Prevention. August 2020. <https://www.maine.gov/dep/spills/topics/pfas/PFOS-Action-Levels-for-Beef-Derivation-Memo-08.04.20.pdf>.

MDH 2024a. Toxicological Summary for: Perfluorooctanoate. Minnesota Department of Health. January 2024. <https://www.health.state.mn.us/communities/environment/risk/docs/guidance/gw/pfoa2024.pdf>.

MDH 2024b. Toxicological Summary for: Perfluorooctane sulfonate. Minnesota Department of Health. January 2024. <https://www.health.state.mn.us/communities/environment/risk/docs/guidance/gw/pfos.pdf>.

Nickerson, A., Rodowa, A.E., Adamson, D.T., Field, J.A., Kulkarni, P.R., Kornuc, J.J., Higgins, C.P., 2021. Spatial Trends of Anionic, Zwitterionic, and Cationic PFASs at an AFFF-Impacted Site. Environ. Sci. Technol. 55, 313–323. https://doi.org/10.1021/acs.est.0c04473

Mikkonen et al. 2023a. Spatio-temporal trends in livestock exposure to per- and polyfluoroalkyl substances (PFAS) inform risk assessment and management measures. Environmental Research 225: 115518. <https://doi.org/10.1016/j.envres.2023.115518>.

Mikkonen et al. 2023b. Dynamic exposure and body burden models for per- and polyfluoroalkyl substances (PFAS) enable management of food safety risks in cattle. Environment International 180: 108218. <https://doi.org/10.1016/j.envint.2023.108218>.

Miller, R.O., R. Gavlak, D. Horneck. 2013. Particle Size Analysis (Hydrometer). p. 95-98. Soil, Plant, and Water Reference Methods for the Western Region. 4th Ed.

MPCA 2024. PFAS ambient background concentrations. Minnesota Pollution Control Agency. March 2024. <https://www.pca.state.mn.us/sites/default/files/tdr-g1-25.pdf>.

MSU 2007. Corn Stover Harvesting. Michigan State University Extension – Reprinted from Cattle Call, 2007, Vol 12, Iss 2. <https://www.canr.msu.edu/uploads/236/58572/CornStoverHarvesting.pdf>.

MSU 2011. Understanding how hay dries in the field. Michigan State University Extension. March 2011. <https://www.canr.msu.edu/news/understanding_how_hay_dries_in_the_field#:~:text=Warm%20air%20temperature%20and%20low,move%20up%20into%20the%20swath>.

Page, A. L., Miller, R. H., & Keeney, D. R. (1982). Methods of soil analysis, part II. *American Society of Agronomy, Madison, WI*.

Sepulvado, J.G., Blaine, A.C., Hundal, L.S., Higgins, C.P., 2011. Occurrence and Fate of Perfluorochemicals in Soil Following the Land Application of Municipal Biosolids. Environ. Sci. Technol. 45, 8106–8112. https://doi.org/10.1021/es103903d

UMN 2018. Plan your forage supply for summer grazing. University of Minnesota Extension. <https://extension.umn.edu/forage-harvest-and-storage/plan-your-forage-supply-summer-grazing>.

USEPA 2024. PRG User’s Guide. U.S. Environmental Protection Agency. <https://epa-prgs.ornl.gov/radionuclides/users_guide.html>.

Vestergren et al. 2013. Bioaccumulation of perfluoroalkyl acids in dairy cows in a naturally contaminated environment. Environmental Science and Pollution Research 20: 7959-7969. <https://doi.org/10.1007/s11356-013-1722-x>.

Xue, J., Verstraete, W., Ni, B. J., Giesy, J. P., Kaur, G., Jiang, D., ... & Uchida, Y. (2025). Rethink biosolids: Risks and opportunities in the circular economy. *Chemical Engineering Journal*, *510*, 161749.

Yoo et al. 2011. Quantitative Determination of Perfluorochemicals and Fluorotelomer Alcohols in Plants from Biosolid-Amended Fields using LC/MS/MS and GC/MS. Environ. Sci. Technol. 45(19): 7985-7990. <https://doi.org/10.1021/es102972m>.
